# The Triangle Relationship Between Long Noncoding RNA, RIG-I-like Receptor Signaling Pathway, and Glycolysis

**DOI:** 10.3389/fmicb.2021.807737

**Published:** 2021-11-30

**Authors:** Zhihua Ren, Yueru Yu, Chaoxi Chen, Dingyong Yang, Ting Ding, Ling Zhu, Junliang Deng, Zhiwen Xu

**Affiliations:** ^1^Key Laboratory of Animal Disease and Human Health of Sichuan Province, College of Veterinary Medicine, Sichuan Agricultural University, Chengdu, China; ^2^College of Life Since and Technology, Southwest Minzu University, Chengdu, China; ^3^College of Animal Husbandry and Veterinary Medicine, Chengdu Agricultural College, Chengdu, China

**Keywords:** glycolysis, lncRNA, antivirus, innate immunity, RIG-I/MAVS

## Abstract

Long noncoding RNA (LncRNA), a noncoding RNA over 200nt in length, can regulate glycolysis through metabolic pathways, glucose metabolizing enzymes, and epigenetic reprogramming. Upon viral infection, increased aerobic glycolysis providzes material and energy for viral replication. Mitochondrial antiviral signaling protein (MAVS) is the only protein-specified downstream of retinoic acid-inducible gene I (RIG-I) that bridges the gap between antiviral immunity and glycolysis. MAVS binding to RIG-I inhibits MAVS binding to Hexokinase (HK2), thereby impairing glycolysis, while excess lactate production inhibits MAVS and the downstream antiviral immune response, facilitating viral replication. LncRNAs can also regulate antiviral innate immunity by interacting with RIG-I and downstream signaling pathways and by regulating the expression of interferons and interferon-stimulated genes (ISGs). Altogether, we summarize the relationship between glycolysis, antiviral immunity, and lncRNAs and propose that lncRNAs interact with glycolysis and antiviral pathways, providing a new perspective for the future treatment against virus infection, including SARS-CoV-2.

## Introduction

Aerobic glycolysis refers to the glycolytic reaction in cells even under aerobic conditions, generating large amounts of lactate. This effect initially occurs in tumor cells and is called the “Warburg” effect. Many studies have reported that aerobic glycolysis occurs in microglia (Cheng et al., 2021a; Wang et al., 2021a), NK cells, and monocytes under different pathological conditions (Cheng et al., 2016; Sheppard et al., 2021) and even virus-infected macrophages (Wu et al., 2021). It is found that the serum and neutrophils of SARS-CoV-2 patients contain high lactate content, which is caused by elevated glycolysis (McElvaney et al., 2020; Jia et al., 2021). As a vital link in glucose metabolism, glycolysis provides intermediates for the biosynthesis of macromolecules during energy production (Locasale and Cantley, 2011; Pearce et al., 2013). Viruses can utilize glycolysis’s energy and molecular precursors for their infection and replication (Passalacqua et al., 2019; Mansouri et al., 2020). *In vitro* and *in vivo* experiments during viral infections such as SARS-CoV-2 have found that increased aerobic glycolysis will cause inflammation and even lead to cytokine storms, resulting in increased viral replication (Thai et al., 2014; Zhu et al., 2021a). Recently, it has been shown that SARS-CoV-2 infection of human monocytes upregulates glycolytic genes, promoting virus replication and the expression of proinflammatory factors (Codo et al., 2020).

Long noncoding RNAs (lncRNAs) are noncoding RNAs that are widespread in various eukaryotes and are more than 200 nucleotides (nt) in length (Marques and Ponting, 2009; Liu et al., 2018). LncRNAs not only regulate the glycolytic network but also regulate multiple regulatory points of antiviral innate immune pathways. The regulation of glycolysis by lncRNA is through the direct regulation of glycolytic rate-limiting enzymes and lactate or indirect regulation of glycolytic upstream genes and kinases. After many studies, it has been found that lncRNAs can regulate antiviral immunity in many kinds of virus infection. On the one hand, the host regulates antiviral innate immunity by targeting lncRNAs (Khatun et al., 2021). On the other hand, the virus suppresses the immune response by lncRNA (Carnero et al., 2016). Strikingly, in peripheral blood mononuclear cells (PBMCs) from SARS-CoV-2 patients, the lncRNA regulatory network showed significant downregulation of interferon-stimulated genes (ISGs) and IFN-I responses (Zheng et al., 2020).

Retinoic acid-inducible gene I-mitochondrial antiviral signaling protein (MAVS) is a major pathway of antiviral innate immunity. The binding of the SARS-CoV-2 N protein to the RIG-I DExD/H domain inhibits RLR-mediated interferon production (Chen et al., 2020). MAVS is a bridge between antiviral innate immunity and glycolysis in the RIG-I-like receptors (RLR) signaling pathway, and Hexokinase 2 (HK2)–MAVS and lactate–MAVS interactions play an important role in antiviral immune responses. During the viral infection, RIG-I-MAVS hijacks HK2-MAVS, thus impairing the normal glycolysis process. However, overexpression of lactate dehydrogenase A (LDHA)-associated lactate can repress MAVS’s subsequent antiviral innate immunity (Zhang et al., 2019b).

Study has found that lncRNAs cluster in glycolysis, as revealed by bioinformatics analysis (Ju et al., 2021). Thus, long noncoding RNAs may also inhibit viral replication by disrupting HK2, voltage-dependent anion channel (VDAC), and MAVS ternary complex to inhibit glycolysis in viral infection. By reviewing the pairwise relationship among glycolysis, antiviral innate immunity, and lncRNA, we propose that lncRNA may affect viral replication by regulating glycolytic flux. The above insight additionally supplies a hypothesis that lncRNAs participate in the interaction of SARS-CoV-2 associated antiviral innate immunity and glycolysis regulation.

## Glycolysis

Glycolytic metabolic enzymes and glucose transporters (GLUTs) regulate glycolysis. Fourteen isoforms of GLUTs transport glucose across the cell membrane to the intracellular space along a concentration gradient (Nomura et al., 2015). Upregulation of GLUT expression and density and accelerated glucose uptake can promote glycolysis (Liu and Gan, 2016; Ryu et al., 2021). Metabolic enzymes in glycolysis [HK, LDHA, and pyruvate kinase (PKM)] and lactate have regulatory effects on glycolysis.

### Key Metabolic Enzymes of Glycolysis

Hexokinase is a primary glycolytic rate-limiting enzyme that catalyzes the initial glucose metabolism step by phosphorylating glucose. There are four isoforms of HK, of which HK2 is the form that is more expressed and regulated in tissues (Roberts and Miyamoto, 2015). HK binds to the mitochondrial outer membrane and interacts with VDACs (DeWaal et al., 2018). HK can be allosterically inhibited and released from mitochondria by the catalytic product glucose-6-phosphate (G6P; Wilson, 2003). The massive dissociation of HK2 induces cell death (Smeele et al., 2011). Knockdown of HK2 inhibits glycolysis while inhibiting mammalian target of rapamycin (mTOR; DeWaal et al., 2018).

Pyruvate kinase converts phosphoenolpyruvate (PEP) to pyruvate. The expression and low enzymatic activity of pyruvate kinase are essential for lactate as a glucose metabolite (Tamada et al., 2012). Pyruvate kinase M2 (PKM2) accelerates the production of GLUT and LDHA by upregulating the expression of the C-MYC gene and further promoting aerobic glycolysis (Yang and Lu, 2013).

Lactate dehydrogenase A, as the terminal enzyme in aerobic glycolysis, converts pyruvate to lactate and is accompanied by the regeneration of NADH to NAD^+^, which is essential for maintaining glycolytic flux. Overexpression of LDHA and pyruvate dehydrogenase kinase (PDK) prevents pyruvate from entering the TCA cycle (Everts et al., 2014). Despite the proinflammatory effects of glycolysis, lactate production through hypoxia–lactate axis can upregulate the expression of macrophage genes such as Tgfb and Il10 and M2-like markers, such as Vegf, Mg1-1, Mgl-2, and CD206, which have the effect of reducing inflammation (Ivashkiv, 2020).

### Upstream Pathway of Glycolysis

Mammalian target of rapamycin is an influential node in the transition from aerobic phosphorylation to glycolysis and is a key metabolic regulator that promotes glycolysis in multiple immune cells, including T cells, B cells, dendritic cells, macrophages, neutrophils, mast cells, and natural killer cells (Xu et al., 2012). mTOR appears as two distinct protein complexes and is divided into mTOR Complex1 and 2 (mTORC1 and mTORC2; Edinger et al., 2003). Activation of mTORC1 signaling encodes nearly every step of glycolysis of gene expression (Duvel et al., 2010). MTORC2 can phosphorylate Akt Ser 473 to maintain Akt activity, increase the binding of HK2 to mitochondria, and promote glycolysis, while HK2 binding to mitochondria is feedback inhibited by G-6P (Hagiwara et al., 2012; Roberts et al., 2013; Roberts and Miyamoto, 2015; Xue et al., 2015).

Currently, the more reported glycolytic pathways are AKT/mTOR or adenosine 5′-monophosphate (AMP)-activated protein kinase (AMPK)/mTOR. Akt phosphorylates tuberous sclerosis complex 2 (TSC2) to activate mTORC1. TSC is a heterologous complex composed of TSC1, TSC2, and TBC1D7, a key factor in regulating mTORC1 activity. TSC2 acts as a GTPase activating protein (GAP) that phosphorylates small GTPase Rheb to inactivate it, activating mTORC1 (Condon and Sabatini, 2019). Akt is involved in glycolysis upregulated by 6-phosphofructo-2-kinase/fructose-2,6-biphosphatase isoenzymes 3/4 (PFKFB3 and PFKFB4), whereas knockdown of Akt decreases lactate accumulation in cells (Houddane et al., 2017).

Adenosine 5′-monophosphate (AMP)-activated protein kinase is a heterotrimeric complex upstream of mTORC1, and its activation leads to the repression of anabolic processes. AMPK, as a cellular energy sensor, signals back to the cell by sensing AMP/ATP ratio and is a link between host cells and mitochondria (Gowans et al., 2013; Lin and Hardie, 2018). AMPK activates TSC2 by phosphorylation, which inhibits mTORC1 function, whereas Akt decreases the AMP/ATP ratio to maintain high levels of ATP, which inhibits the AMPK-mediated phosphorylation and the activation of TSC2 (Wullschleger et al., 2006).

The regulation of HIF and c-Myc also has a certain promotion effect on glycolysis. Hypoxia-inducible factor (HIF-1α) can directly upregulate the GLUT-1 and participate in glycolytic metabolic enzymes to increase the glycolytic flux, such as hexokinase, pyruvate kinase, and lactate dehydrogenase (Seagroves et al., 2001). What is more, HIF trans-activates PDK1 to phosphorylate pyruvate dehydrogenase (PDH) and blocks the conversion of pyruvate to acetyl-CoA, which in turn promotes glycolysis (Kim et al., 2006). Activation of AKT/mTOR can stimulate elevated HIF-1α protein levels (Kaidanovich-Beilin and Woodgett, 2011). Phosphorylation and activation of AMPK inhibit HIF1α and NFκB by the deacetylation of regulating proteins (Kim, 2018). As a transcription factor, C-Myc stimulates the same glycolytic genes and enhances the glycolytic pathway as HIF-1. C-Myc increases the target gene expression of GLUTs *via* pyruvate kinase and LDHA, allowing glucose-derived lactate efflux (Osthus et al., 2000).

## Lncrna’s Role in Glycolysis

Long noncoding RNAs regulate glycolysis mainly through three aspects. (1) lncRNAs regulate glycolysis by regulating metabolic pathways, including activation of the AKT/mTOR signaling pathway, c-Myc, and miRNA sponge action; (2) LncRNA regulates glucose-related metabolic enzymes, genes, including GLUTs, HK, and LDHA; and(3) LncRNAs regulate glycolysis through epigenetic reprogramming, including histone acetylation and DNA methylation regulation.

**Figure 1:**
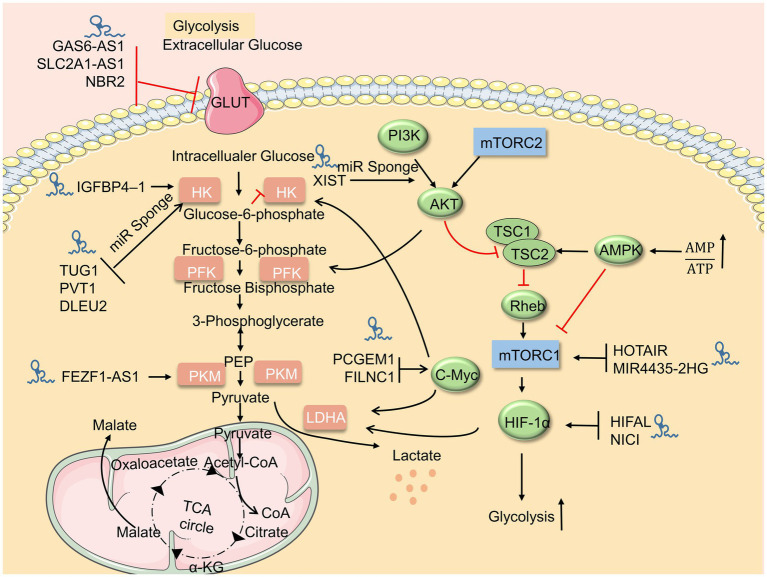
LncRNAs regulate the glucose metabolism. Upstream metabolic pathways interact downstream glycolysis sites. Many lncRNAs regulate glycolysis *via* glycolysis sites, adenosine 5′-monophosphate (AMP)-activated protein kinase (AMPK)/mammalian target of rapamycin (mTOR), and AKT/mTOR pathway.

At present, studies on the regulation of glycolysis by LncRNA mainly focus on different tumor cells. Many lncRNAs can promote the proliferation and invasion of malignant tumor cells, but many lncRNAs also play an inhibitory role in tumor cells. These studies suggest that lncRNAs may act as cellular metabolic regulatory points and then provide new ideas for disease treatment. It is worth noting that lncRNAs, in addition to directly regulating glycolysis, also act as competing endogenous RNAs (ceRNAs) of microRNAs to become mediators of metabolic reorganization for immune cells (Table 1; Figure 1).

**Table 1:** Long noncoding RNA (LncRNA) links glycolysis.

Item	LncRNA name	Tissue/cell	Targets	Regulation sites	Links to glycolysis
Enzyme	GAS6-AS1	HBE and LUAD cell lines (A549, H1299, PC9, and H1975)	E2F1	GLUT1 ↓	↓ Luo et al., 2021
	SLC2A1-AS1	Human nontumor liver cell line HL-02 Human HCC cell lines MHCC97-H, Huh7, HepG2, and Hep3B	Forkhead box M1 ↓	GLUT1 ↓	↓ Shang et al., 2020
	NBR2			GLUT1 ↓	↓ (under the condition of depleting lnc NBR2; Liu and Gan, 2016)
	IGFBP4–1	Human lung adenocarcinoma cancer cell lines (A549, PC-9, and GLC-82)		HK2, PDK1, and LDHA ↑	↑ (under the condition of overexpressing lnc-IGFBP4-1; Yang et al., 2017)
CeRNA	TUG1	Hepatocellular carcinoma cell	miR-455-3p ↓	AMPKβ2 ↓, HK2 ↑	↑ (Lin et al., 2018)
	PVT1	Gallbladder cancer (GBC) tissue	miR-143 ↓	HK2 mRNA and protein level ↑	↑ (Chen et al., 2019)
	DLEU2	HEC-1, HEC-50, HHUA, Ishikawa, KLE cells, and endometrial epithelial cell line	miR-455 ↓	HK2 ↑	↑ (Dong et al., 2021)
	XIST	Human glioblastoma cell line (U87MG, U251, U343, Hs683, LN215, and A17224); Primary normal human astrocytes (NHAs) HA1800	miR-126 ↓	IRS1/PI3K/Akt pathway ↑	↑ (Cheng et al., 2020)
Signaling pathway	HOTAIR	HepG2, SMMC-7721, Hep3b, Huh7, and Bel-7402 cells	mTOR ↑	GLUT1 ↑	↑ (Wei et al., 2017)
	FEZF1-AS1	Colorectal cancer cell lines LoVo, Caco2, HT29, HCT8, HCT116, and SW480	STATE3 ↑	Pyruvate kinase 2 (PKM2) ↑	↑ (Bian et al., 2018)
	HIFAL	Breast cancer cell lines	HIF-1α ↑	Propyl hydroxylation of PKM2 ↑ propyl hydroxylation of PKM2	↑ (Zheng et al., 2021)
	NICI	Human PTCs, HeLa, MCF-7, Hep3B, HepG2, HEK293T, T47D, and HT1080 cell	HIF-1α ↑	SLC2A3 (coding for GLUT3) ↑	↑ (Lauer et al., 2020)
	PCGEM1	Prostate cancer. Cell lines LNCaP, PC3, and HEK293T	c-Myc	Lactate ↑	↑ (Hung et al., 2014)
Gene	MIR4435-2HG	Primary myeloid dendritic cells (mDCs)	mTORC1 (RPTOR gene locus) ↑		↑ (Hartana et al., 2021)
	FILNC1	Renal cancer cells	c-Myc ↑	Lactate ↑	↑ (under the condition of knockdown Lnc FILNC1; Xiao et al., 2017)
	LINC01559, UNC5B-AS1	Pancreatic cancer cell lines (AsPC-1, BxPC-3, Capan-1, PANC-1, and SW1990)	Glycolysis associated genes variations (MYC, GATA6, and FGFR1, IDO1, and SMADA) and mutations (KRAS, SMAD4, and RNF43)		↓ (Zhu et al., 2021b)

## Glycolysis and Innate Immunity

Glycolysis is tightly associated with immune cells, trained immunity, and the antiviral pathway as a critical part that supplies energy. Viruses facilitate cellular glycolysis by using signaling pathways upstream of glycolysis, key enzymes, and mitochondrial function, which achieves viral replication and evades innate immunity. The interaction of MAVS with HK2 and lactate communicates the relationship between immunity and metabolism.

### Glycolysis and Immune Cells

**Figure 2:**
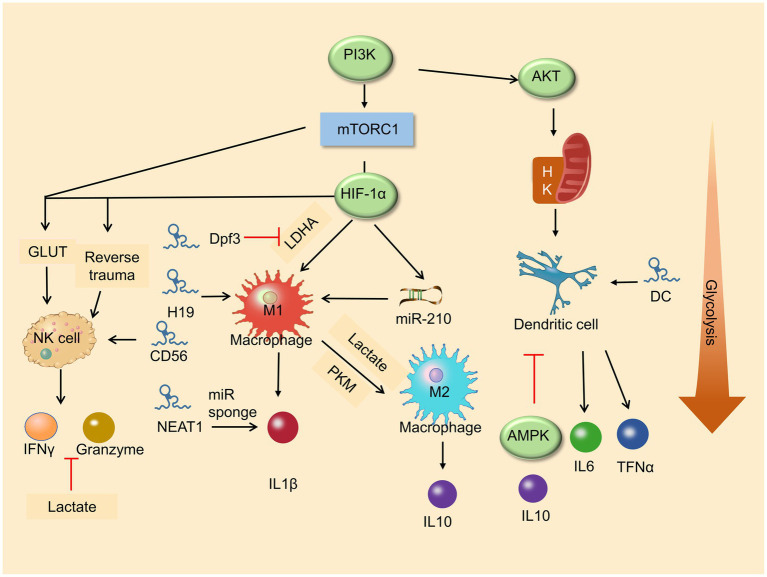
Roles of metabolic pathway and lncRNA in immune cell differentiation. Glycolysis enhances macrophage M1 polarization and promises the function of NK cells. LncRNAs play a vital role in immune cells in differentiation and development.

Increased glycolysis is a marker of activation of many immune cells and results in changes in various immune cell functions (e.g., natural killer cells, macrophages, and dendritic cells), which indicates that glycolysis is essential in innate immune function (O’Neill et al., 2016; Buck et al., 2017; Figure 2).

Nk cells are innate lymphocytes involved in controlling early intracellular pathogen infection and produce IFN-γ by recognizing surface receptor activation of target cells (Vivier et al., 2008). The function of NK cells to produce IFN-γ and granzyme B is achieved by mTOR-mediated upregulation of glucose uptake and glycolysis rate, while mTOR activity is controlled by NK cells developmental signals, inflammatory signals and partly controlled by PI3K (Donnelly et al., 2014; Marcais et al., 2014; Viel et al., 2016). Similarly, cytokines (e.g., IL-10) target mTOR to activate NK cells to upregulate nutrient transport receptors (e.g., glucose transport receptors and amino acid transport receptors), promoting glycolysis and enhancing NK cells cytotoxicity and producing IFN-γ (O’Brien and Finlay, 2019; Wang et al., 2021b). In addition, cytokine-induced glycolytic flux, IFN-γ, and granzyme B secretion were reduced without affecting sterol regulatory element-binding protein (SREBP) activity in natural killer cells (Assmann et al., 2017). Moreover, Seon Ah Lim found that NK injury could be reversed by the HIF-1α-mediated shift from oxidative phosphorylation to glycolysis (Lim et al., 2021). However, lactate is an influential factor that inhibits the function and survival of NK cells (Brand et al., 2016).

NK cell function is closely related to aerobic glycolysis; other immune cells are similarly associated with glycolysis. The metabolic shift from oxidative phosphorylation to aerobic glycolysis is an important marker of activating macrophages and dendritic cells and an altered metabolism involved in the signal-directed program of pro-inflammation or anti-inflammation (O’Neill and Hardie, 2013). HIF-1α is a master regulator of aerobic glycolysis and plays a crucial role in macrophage polarization to the M1 phenotype associated with inflammation, and HIF-1α stabilization promotes IL-1α production (Tannahill et al., 2013). Similarly, HIF-1α is upregulated in SARS-CoV-2 patients and promotes macrophage inflammatory responses (Zhu et al., 2021a). HIF-1α can also induce miR-210 to shift macrophages to a proinflammatory state while knocking down miR-210 limits the cytokine storm (Virga et al., 2021). Nevertheless, the complex formation between HIF-1α and PKM2 inhibits the expression of HIF-1α and IL-1β and promotes the expression of IL-10, in turn leading to a shift in the macrophage phenotype from proinflammatory M1 to pro-reparatory M2 (Palsson-McDermott et al., 2015). Lactate can counteract the inflammatory effects of HIF-1α and glycolytic metabolites and increase anti-inflammatory genes’ expression (Ivashkiv, 2020). More than this, HIF-1α-mediated lactate induces M2-like polarization of macrophages (Colegio et al., 2014). Consistently, lactate produced by c-Myc-promoted glycolysis inhibits the production of proinflammatory cytokines in early polarized M1 macrophages (Bae et al., 2021). Increased glycolysis of dendritic cells results in IL-6 and TNF-α production (Cheng et al., 2021b). This increase in glycolysis is conducted by Akt driven by the kinases TBK1 and IKKε, which directly promotes the association of the glycolytic enzyme HK-II with mitochondria (Everts et al., 2014). In addition, glycolysis in dendritic cells can also be activated through PI3K/Akt, inhibiting AMPK and IL-10 (Krawczyk et al., 2010). However, inhibition of glycolysis decreases dendritic cell activation and longevity.

### Glycolysis and Trained Immunity

Glycolysis plays a crucial role not only in regulating immune cell function but also in training immunity. Trained immunity refers to an enhanced host defense response when subjected to homogenous or heterologous stimulation after receiving an immune stimulation for the first time. This immune memory mainly occurs in immune cells, including monocytes, macrophages, and NK cells (Netea et al., 2011). Trained immunity is regulated by epigenetic reprogramming and cellular metabolic reprogramming. For example, peripheral monocytes trained with the BCG vaccine have increased H3K4me3 histone modifications associated with promoters of TNFα, IL6, and TLR4 genes, leading to transcriptional activation of proinflammatory cytokines (Covian et al., 2019). In addition, the process of trained immunity by β-glucan-trained monocytes showed increased glucose consumption, lactate production, and NAD +/NADH ratio. This increase in glycolysis depends on the dectin-1/Akt/HIF1α/mTOR axis while inhibiting Akt, mTOR, or HIF1α inhibits the training immunity of monocytes (Cheng et al., 2014). Other studies have also shown that mTOR-dependent HIF1α is a prerequisite for metabolic reprogramming during trained immunity (Bekkering et al., 2018).

Epigenetic reprogramming and cellular metabolic reprogramming also present interactions during trained immunity. Increased glycolysis promotes cytokine production, and cytokine production is achieved by histone modification of cytokine gene promoters. Metabolic intermediates can be served as substrates for epigenetic reprogramming enzymes. For example, the sirtuin family of histone deacetylases (HDACs) depends on the intracellular NAD+ concentration. Moreover, increased expression of glycolytic enzymes is mediated through histone modification effects (Riksen and Netea, 2021).

Similarly, the control of macrophage glycolysis on histones is fundamental to trained immunity, and the inhibition of glycolysis leads to repressing trained immunity (Ieronymaki et al., 2019; Keating et al., 2020). The interaction between epigenetic mechanisms and aerobic glycolysis does not only occur in trained immunity. LDHA in Th1 cells promotes the expression of IFN-γ through histone acetylation (H3K9Ac), which occurs in LDHA-mediated aerobic glycolysis (Peng et al., 2016).

### The Association Between Glycolysis and Antiviral Innate Immunity

#### Virus Recognition

Innate immunity is a non-specific immunity against foreign pathogens and plays a critical role in protecting higher organisms from pathogenic infections. Infection of host cells by viral pathogens in the antiviral response is recognized by pattern recognition receptors (PRRs). The RLRs are the principal defense cascade molecules of sensing RNA viruses and have cross talk with the recognition mechanism of DNA viruses (Xing et al., 2012; Cai et al., 2021). RIG-I consists of a DExD/H-box helicase-like domain-containing ATPase and translocase activity, a repressor regulatory domain (RD) at the C-terminal, and two caspase-associated recruitment domains (CARD) at the N-terminal (Takeuchi and Akira, 2010; Schlee and Hartmann, 2016). When the CTD of RIG-I recognizes 5′ triphosphate-containing double-stranded (DS) viral RNA, the conformation of RIG-I protein is changed that k63 linked E3 ligase polyubiquitinated at different sites (Sanchez-Aparicio et al., 2017). Hence, the CARD domain of RIG-I binds to the CARD domain of the downstream adaptive molecule MAVS to induce its activation, and MAVS redistributes and accumulates on the mitochondrial surface to activate IRF3 in response to viral infection (Hou et al., 2011). MAVS phosphorylates IRF3/IRF7 and NF-kB by activating TBK1 kinase and IKKε, which drives their nuclear translocation, and then activating IRF3 dimer, NF-kB, and AP-1 transcription factors leading to IFN-I transcription and proinflammatory cytokine production. The secreted IFN-I activates IFNAR, leading to phosphorylation and nuclear translocation of STAT1, thereby enabling the expression of IFN-stimulated genes (ISGs) with extensive antiviral function (Stark and Darnell, 2012). MAVS deletion produces neither activation of NF-κB- and IRF3-responsive viruses, nor IFN-I and proinflammatory factors in RIG-I-mediated antiviral immune responses. The above indicates that MAVS is the sole adaptor downstream of RIG-I (Kumar et al., 2006; Sun et al., 2006). MAVS co-localizes with the mitochondrial outer membrane protein Bcl-xL at the mitochondrial outer membrane, while mislocalization of MAVS leads to decreased activity and impaired antiviral function (Seth et al., 2005). Moreover, The TRIM family’s ubiquitination generally accompanies post-transcription regulation of MAVS (e.g., TRIM21, 25, 29, and 31) in response to virus infection (Oshiumi et al., 2010; Xing et al., 2016, 2018; Ren et al., 2020). SARS-CoV-2 nonstructural protein5 (nsp5) cleaves RIG-I and promotes MAVS degradation to evade innate immunity (Liu et al., 2021b).

#### Glycolysis and Antivirus

Metabolic pathways and antiviral pathways are closely linked. In Huh7 cells, the Akt-mTOR axis was activated at the early stage of SARS-CoV-2 infection to promote virus replication, while the use of the AKT inhibitor MK-2206 significantly reduced the virus replication and infection capacity. Exposing cells to high glucose significantly increased viral infection, whereas viral replication was significantly reduced after using glycolytic inhibitors, and viral infection was restored after adding exogenous ATP (Kohio and Adamson, 2013). Other studies have shown that cellular glycolytic flux is significantly increased during viral infection and is a major source of ATP (Rezinciuc et al., 2020), as viral entry initiates glycolysis, and viral replication amplifies this metabolic change. Glycolysis provides the required metabolic fuel for virus replication, while inhibition of glycolysis reduces viral titers (Smallwood et al., 2017). As a crucial protein for viral replication, coronavirus nonstructural protein 13 (SCV nsp13) promotes synergistic translocation in the unwinding of duplex RNA in high concentrations of ATP (Jang et al., 2020).

Increased glycolysis contributes to proinflammatory factors’ production and viral replication (Chi et al., 2018; Erlich et al., 2020), while low-flux glycolysis enables RLR-mediated IFN-I production, enhancing viral clearance capacity. Zhang et al. (2019b) found that PEP, pyruvate, and lactate were downregulated during the initial phase of RLR-mediated type I interferon production, indicating impaired glycolysis. Increased production of IFN-β and IL-6 and reduced viral replication were found in both Sendai virus (SEV) and herpes stomatitis virus (VSV)-infected mice and cells under the effect of 2-deoxy-ᴅ-glucose (2-DG; Zhang et al., 2019b). In addition, it was shown by using galactose medium and 2-DG that inhibition of glycolysis can repress HCV from cell to supernatant release (Yu et al., 2021). While glycolysis may contribute to the inflammatory storm (Wang et al., 2020b; Virga et al., 2021), the absence of glycolysis can lead to the loss of antiviral function in NK cells. Sheppard et al. (2021) found that LDHA-mediated aerobic glycolysis is a hallmark of NK cell activation. However, NK cells are more susceptible to MCNV infection in the absence of LDHA, caused by the decreased value-added rate and defective cytotoxicity of NK cells deficient in LDHA (Sheppard et al., 2021). Consistently, in early NK cells activated by IL-2/IL-12, mTOR1 significantly upregulated c-Myc and increased the rate of glycolysis, whereas NK cells lacking the c-Myc transcription factor downregulated IFNγ and cytolytic molecule granzyme B (Loftus et al., 2018). 2-DG in NK cells infected by mouse cytomegalovirus (MCMV) inhibits glycolysis, which in turn leads to decreasing expression of cytotoxic proteins and altering the adhesion of NK cells to target conjugates (Mah et al., 2017).

Glycolytic rate-limiting enzymes can represent increased glycolysis during viral infection. Elevated levels of HIF-1a protein and its transcriptional activity as measured by GLUT-1, PKM2, and LDHA were found in SARS-CoV-2-infected monocytes, resulting in increased glycolytic flux (Codo et al., 2020). This increase in glycolysis is necessary for SARS-CoV-2 replication. Ramiere et al. (2014) found that the hepatitis C virus (HCV) can increase HK2 enzyme activity in host cells through the viral protein NS5A, showing increased glucose consumption and lactate secretion. LDHB catalyzes the conversion of lactate and NAD+ to pyruvate, NADH, and H+. Fan et al., 2020 found that classical swine fever virus (CSFV) infection affects lactate and pyruvate content in PG-15 cells *via* LDHB. LDHB inhibits CSFV replication through the NFκB signaling pathway to repress the replication of CSFV (Fan et al., 2020).

Adenosine 5′-monophosphate (AMP)-activated protein kinase inhibits inflammation and viral replication, mediated by the inhibition of glycolysis (O’Neill and Hardie, 2013; Singh et al., 2020). ZIKV infection caused a time-dependent reduction in the active phosphorylated state of AMPK and its downstream target acetyl-CoA carboxylase. AMPK activation inhibits virus-induced glycolysis, enhances genes’ expression with antiviral properties (e.g., IFN, OAS2, ISG15, and MX1), and suppresses inflammatory mediators (e.g., TNF-a and CCL5; Singh et al., 2020). In VSV-infected mouse macrophages or mouse embryonic fibroblasts (MEFs), AMPK is also found to promote innate immunity by stimulating the Stimulator of Interferon Genes (STING) to upregulate IFN (Prantner et al., 2017). However, it has been shown that activating and promoting the expression of the catalytic subunit AMPKα2 in fibroblasts infected by HCMV promote glycolysis and induce an environment conducive to viral infection (Dunn et al., 2020).

Hypoxia-inducible factor-1 and c-Myc regulate glycolytic flux during viral infection. Under normoxic conditions, b-catenin specifically interacts with HIF-1α in alveolar macrophages (AMs) infected by respiratory viruses, thus regulating HIF-1α-driven glycolysis to promote excessive inflammation, leading to inhibition of AMs proliferation (Zhu et al., 2021a). Similarly, cellular mitochondrial oxidative phosphorylation is impaired by HCV infection, resulting in upregulation of HIF-1 and consequently glycolysis-related gene expression (Ripoli et al., 2010). Avian reovirus (ARV) structural protein σA was found to inhibit LDHA, upregulated hexokinase, and pyruvate kinase mRNA through HIF-1α to promote ATP production and viral replication (Chi et al., 2018). What is more, c-Myc promotes glycolysis leading to increased ATP production, which promotes mitochondrial biogenesis. Adenovirus e4orf1 infection of mammary epithelial cells induced activation of c-Myc, significantly improved transcription of HK2 and PFKM, and increased glycolysis (Thai et al., 2014). Nasopharyngeal carcinoma (NPC) cells infected by Epstein–Barr virus (EBV) have similar conclusions (Xiao et al., 2014). Upregulation of c-Myc in four DNA tumor viruses, human adenovirus, human papillomavirus, EBV, and Kaposi’s associated sarcoma herpesvirus, affects cellular metabolism (Prusinkiewicz and Mymryk, 2021). Glycolysis and c-Myc expression were increased in DC cells infected with IAV while inhibiting c-Myc activity blocked the increase of glycolysis induced by IAV (Rezinciuc et al., 2020).

Glycolysis and MAVS-RIG-I are each other’s negative regulators, and MAVS communicate the relationship between antiviral immunity and metabolism. This relationship is mainly reflected through HK2-MAVS and lactate–MAVS interaction. On the one hand, glycolysis is impaired during RLR signal activation, and on the other hand, glycolysis inhibits MAVS-TBK1-IRF3 activation and IFN-I production. When RLR triggers MAVS-RIG-I recognition, the binding of MAVS to HK2 switches to binding to RIG-I, resulting in impaired mitochondrial localization and activity of HK2, which in turn impairs subsequent glycolysis (Zhang et al., 2019b), which was also confirmed by Chauhan et al. (2019). In addition, the interaction of HK2 with MAVS requires binding to VDAC.

**Figure 3:**
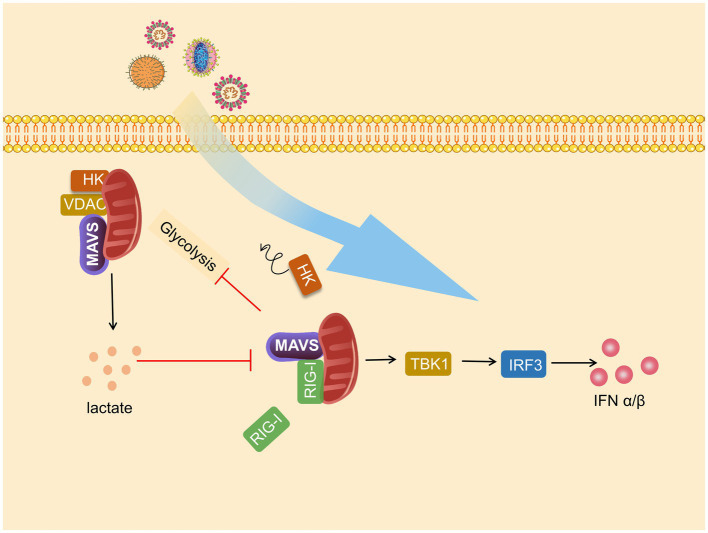
Mitochondrial antiviral signaling protein (MAVS) is a bridge between immunity and glycolysis. Viruses promote hexokinase (HK) activity and lactate production and subsequently impress TANK-binding kinase 1 (TBK1)-IRF3 activation and IFN-I production. Mitochondria HK activity inactivated, and glycolysis is suffered blocks during RIG-I-like receptor (RLR) activation. Lactate negatively regulates RLR-mitochondrial antiviral signaling protein (MAVS) signaling and following reactions.

Furthermore, in the subsequent study, Chao found that HK2 colocalizes with MAVS and interacts with MAVS CARD domains. However, after this interaction was disrupted, HK2 activity and lactate amount decreased (Chao et al., 2019). LDHA-associated lactate has a blocking effect on the MAVS-RIG-I signaling pathway. Lactate specifically binds to the TM structural domain of MAVS to inhibit its mitochondrial localization, impair MAVS accumulation, disrupt MAVS-RIG-I interactions, and thus repress IFN-I production. Knockdown of HK2 enhanced TBK1-TRF3 signaling, promoted cytokine production, and strengthened the inhibitory effect on viral replication, which inhibited the inhibitory effect of lactate on MAVS (Zhang et al., 2019b). Similarly, Zhou et al., 2021 found that lactate plays a proviral role in interfering with IFN-β expression and helps HBV-mediated immune escape on virus-infected hepatocellular carcinoma cells and mice (Figure 3).

Moreover, miR-33/33* was found in macrophages infected with vesicular stomatitis virus (VSV) to inhibit MAVS accumulation by targeting AMPK, thereby inhibiting RIG-I signaling (Liu et al., 2021a). Other studies have also shown that AMPK decentralizes MAVS distribution in mitochondria and inhibits proinflammatory factors (IFN-β and IL-6; Hanada et al., 2020). Therefore, we suggest that AMPK may inhibit MAVS accumulation by regulating HK2 or lactate, but the specific mechanism needs further experimental proof.

### Mitochondria Play a Vital Role in Innate Immunity

Not only MAVS is anchored on the mitochondrial membrane surface, but HK2 is also on the mitochondrial membrane surface. Mitochondria are, therefore, the site of communication between antiviral innate immunity and metabolism. As a multi-protein complex, NLRP inflammasomes can promote the maturation and secretion of downstream proinflammatory cytokines (e.g., IL-1β and IL-18), specifically dependent on VDACs (Zhou et al., 2011). mTORC1-HK-dependent glycolysis is required for NLRP3 inflammasome activation in response to proinflammatory stimuli (Moon et al., 2015). HK interacts with VDAC to localize to the outer mitochondrial membrane, and dissociation of HK induces NLRP3 activation and IL-1β production (Wolf et al., 2016), leading to mitochondrial damage and mitochondrial ROS (mtROS) production (Mills et al., 2017). MtROS formation occurs downstream of the mTOR-cytoROS-HIF1α feedback loop and can induce HIF-1α to promote inflammatory signaling in macrophages, but inhibition of mtROS represses the increase in lactate production (Sohrabi et al., 2018; Silwal et al., 2021). MtROS drives the polymerization of MAVS and the generation of type-I interferon (Buskiewicz et al., 2016). MAVS promotes the recruitment and activation of NLPR3 to mitochondria (Subramanian et al., 2013). In contrast, viruses can target MAVS to interact with NLRP3 and inhibit NLRP3 activation (Cheung et al., 2020), suggesting that mitochondria are the signaling center that activates antiviral innate immunity and glycolysis. That inflammasome may be involved in MAVS and HK2 interactions. Furthermore, many studies have shown that lncRNAs are characterized by subcellular localization, and this localization role is also included in mitochondria (Wilk et al., 2016).

## Antiviral Innate Immune Effects of Lncrna

### LncRNA Regulates Immune Response and Immune Cell Differentiation

Long noncoding RNA and inflammatory pathways are bidirectionally regulated. Lnc01140 inhibits NF-κB activity and reduces macrophage inflammatory response *via* downregulation of miR-23b (He et al., 2020). TNFα and IL-1β can upregulate LncRNA Lethe *via* NF-κB. In contrast, Lethe interacts with the NF-κB subunit RelA, which inhibits the binding of RelA to target genes (Rapicavoli et al., 2013). Induction of LncRNA DNM3OS overexpression by activated NF-κB in mouse bone marrow-derived macrophages (BMDMS) leads to upregulation of inflammatory and immune response genes under diabetic conditions (Das et al., 2018). In addition, LncRNA MacORIS inhibits the expression of IFNγ-responsive genes in macrophages *via* the JAK2/STAT1 phosphorylation pathway (Zhang et al., 2017). LncRNA GAS5 upregulates mir-544/RUNX3, increasing IFN-γ and NK cell toxicity (Fang et al., 2019). LncRNA expression regulates the immune response and plays a role in developing myeloid and lymphoid cells polarization (Ahmad et al., 2020). Li et al. found that LncRNA H19 enhanced the activation of M1 polarization in Kupffer cells and promoted the recruitment and differentiation of bone marrow-derived macrophages (Li et al., 2020). Lnc-DC promotes the differentiation of dendritic cells and facilitates phosphorylation of STAT3 on tyrosine-705 (Chapman et al., 2014). LncRNA CD56 positively regulates the human NK cell marker CD56, which is involved in NK cell differentiation and development (Zhang et al., 2016). DC-specific lnc-Dpf3 deficiency enables CCR7 to activate the HIF-1α pathway of DC cells and increase glycolysis. However, lnc-DPF3 directly binds to HIF-1α and inhibits transcription of HIF-1α-dependent glycolysis gene LDHA, thereby inhibiting DC glycolysis metabolism and migration (Liu et al., 2019b). In addition, lncRNAs act as ceRNAs in regulating both immune cell differentiation and inflammatory responses (Wang et al., 2019b; Nie and Zhao, 2020).

### LncRNA Regulates Antiviral Pathways and Viral Replication

**Figure 4:**
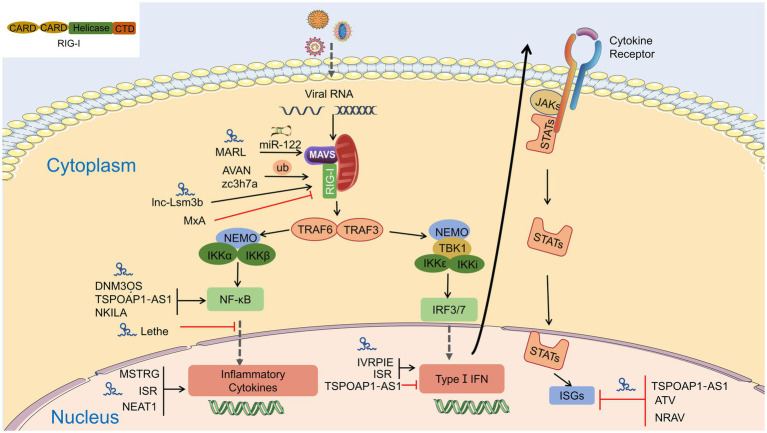
Interaction of lncRNA with RIG-I-MAVS pathway. LncRNAs are regulators in NF-κB and IRFs *via* the RIG-I-MAVS pathway and finally modulate IFN/cytokines. LncRNAs also directly regulate IFNs, cytokines, and even interferon-stimulated genes (ISGs) through Janus Kinases (JAKs)/signal transducer and activator of transcriptions (STATs) pathway.

Target genes of lncRNAs during viral infection are mainly enriched in inflammatory pathways and antiviral signaling pathways, including “NF-κB signaling pathway,” “RIG-I-like receptor signaling pathway,” “Jak-signal transducer and activator of transcription (STAT) signaling pathway,” and “TNF-signaling pathway,” which are finally reflected in the production of interferons and inflammatory cytokines (Gao et al., 2021). LncRNA also regulates epigenetic markers of IFN-encoding chromatin, IFN expression, and susceptibility to viruses (Gomez et al., 2013), expression of inflammatory mediators, ISGs, immune genes (Carpenter et al., 2013; Cui et al., 2014; Zhang et al., 2019a). LncRNAs also act as ceRNAs in regulating innate immunity against viral infection. LncRNA IFITM4P can be used as a target of miR-24-3p to regulate the mRNA levels of interferon-induced transmembrane proteins (IFITM1, IFITM2, and IFITM3) and thereby inhibit influenza A virus (IAV) virus replication *in vitro* (Xiao et al., 2021). Similarly, LncRNA (MARL) found in scleractinian fish infected with Siniperca chuatsi rhabdovirus (SCRV) acts as a ceRNA for miR-122 to enhance the abundance of MAVS protein, thereby promoting MAVS-mediated antiviral responses (Chu et al., 2020). On the one hand, lncRNAs act as positive regulators of antiviral innate immunity and inhibit viral replication (Khatun et al., 2021); on the other hand, the expression of some lncRNAs may facilitate viral infection and replication (Ouyang et al., 2014; Shirahama et al., 2020). Therefore, overexpression or silencing of lncRNAs to control antiviral immune responses may be a future direction in antivirus (Table 2; Figure 4).

**Table 2:** The role of lncRNA in antiviral innate immunity.

LncRNA name	Cell	Virus	Site	Mechanism/regulatory effect	Antivirus function
AVAN	A549, BEAS-2B	IAV	Neutrophil activation, RIG-I, IFN-I, and ISGs ↑	AVAN remodels the FOXO3a promoter region to promote neutrophil chemotaxis and recruitment. AVAN enhanceTRIM25-mediated K63-linked ubiquitination of RIG-I	+(Lai et al., 2021)
IVRPIE	A549, BEAS-2B	IAV	mRNA and protein levels of IFNβ1 and ISGs↑	Histone modification on transcription start site to promote transcript of IFN β1 and ISGs	+(Zhao et al., 2020)
NKILA	HEK293T, TZM-bl	HIV-1	HIV-1 LTR Promoter activity.↓, NF-ĸB-dependent signaling ↑	HIV-1 infection reduced acetylation of histone K27 on the promotor of NKILA to repress expression of NKILA	+(Wang et al., 2020a)
ISR	A549	IAV	RIG-I and NF-κB dependent Signaling ↑	LncRNA ISR suppresses IA V replication and is identified as an ISG gene	+(Pan et al., 2019)
Lnczc3h7a	HEK 293T cells	VSV	Stability of TRIM25-RIG-I complex ↑, MAVS ↑, and IFN-I ↑	Lnczc3h7a facilitates TRIM25-mediated K63-linked ubiquitination of RIG-I	+(Lin et al., 2019)
TSPOAP1-AS1	A549, THP-1	IAV	NK-κB ↑,ISGs ↓, and type I IFN		
MSTRG (silenced)	Porcine ST cells	SVV	IL-10 ↑, TNF-α, IL-1, IL-6, and IL-8 ↓	SVV replication ↓	−(Zhu et al., 2020)
ATV (silenced)	HuH7	ZIKV, NDV, and SeV	IFN β, ISG ↑	Virus replication ↓, RIG-I pathway-negative regulator	−(Fan et al., 2019)
Lnc-Lsm3b	Mouse macrophage RAW264.7 cell line	VSV	RIG-I, IRF3, NF-kB promoter, and IFN-I ↓	Binding of lnc-Lsm3b to CTD and helicase domain of RIG-I restrict RIG-I protein conformation shift, thus making RIG-I lose TRIM25 binding ability, and CARD ubiquitination	−(Jiang et al., 2018)
MxA	MDCK 293T A549 cells	IAV	IFN transcription, RIG-I mediated pathway ↓	MxA form RNA–DNA triplex with the promoter of IFNβ to interfere with the activation of IFNβ	−(Li et al., 2019)
NRAV	A549 cells	IAV	ISGs transcription (IFITM3,MxA) ↓	Histone modification of ISGs to inhibit transcription, IAV replication, and virus production ↑	−(Ouyang et al., 2014)
IPAN	HEK293T-Gluc cells	IAV	Stability of viral RNA polymerase PB1 ↑	Viral RNA synthesis, virus replication ↑	−(Wang et al., 2019a)

SVV, Seneca Valley virus; ZIKV, Zika virus; NDV, newcastle disease virus; VSV, vesicular stomatitis virus; IAV, influenza A virus; SeV, Sendai virus; ISGs, interferon-stimulated genes; LTR, long terminal repeat; A549, human lung adenocarcinoma epithelial cells; BEAS-2B, human bronchial epithelial cells; MDCK, Madin–Darby canine kidney cells; 293T, human embryo kidney cells; “+,” a positive effect associated antiviral functions; and “-,” a negative effect associated antiviral functions.

## The Relationship Between Glycolysis, Lncrna, and Antiviral Innate Immunity

Although many studies have shown that lncRNAs regulate viral replication and proliferation and the antiviral signaling pathways of viral infection are well understood, the mechanism of action of some lncRNAs for antiviral function remains unclear. During viral infection, the rise in aerobic glycolysis provides favorable conditions for viral replication and promotes the secretion of inflammatory factors. MAVS disrupted the mitochondrial localization of HK2, whereas lactate production inhibited MAVS-RIG-I interaction. Therefore, RIG-I-MAVS is an essential pathway of immunity and metabolism.

Long noncoding RNAs can regulate glycolysis in immune cells, and interfere with metabolic pathways and anabolism in virus-infected cells. Studies have shown that the lncRNA MIR4435-2HG in HIV-infected macrophages promote glycolysis by targeting the gene (RPTOR) on mTORC1 that controls glycolysis (Hartana et al., 2021). LncRNAs inhibit viral replication by binding glutathione S-transferase M1 (GSTM1) and blocking the interaction with the kinase TBK1, elevating TBK1 activity and IFN-I production (Wang et al., 2020c). IFN-I-IRF3 axis non-dependent LncRNA-ACOD1 feedback promotes viral replication by promoting the catalytic activity of the metabolic enzyme glutamic oxaloacetic transaminase (GOT2; Wang et al., 2017). In hepatitis C-infected cells, lncRNA promotes adipogenesis, is vital for releasing viral particles, and has a beneficial effect on viral replication (Sharma et al., 2019; Khatun et al., 2021). In addition, in many studies, LncRNA often regulates glycolysis in hepatocellular carcinoma cells (Shang et al., 2020), caused by HBV and HCV infection (Nguyen et al., 2020). A previous study reported that multiple lncRNAs significantly enrich and regulate interaction networks, including metabolic and TNF signaling pathways in infected Porcine delta coronavirus (PDCoV) cells (Liu et al., 2019a). Besides, several lncRNA-associated genes are also found clustering in the glycolytic process in African swine fever virus (ASFV)-infected cells, contributing to ASFV pathogenesis (Ju et al., 2021).

Exploration of the relationship among the three gave us several thoughts. The previous section of this paper showed that LncRNA could act as a regulator of RIG-I, which is necessary for activating downstream TBK1/IRF3 by MAVS (Cai et al., 2017). What is more, MAVS is inhibited by HK2 and lactate, while LncRNA may regulate HK2- or LDHA-dependent lactate production *via* upstream kinase stimulation. Accordingly, the role of miRNA in regulating RIG-MAVS and the function of lncRNA as a ceRNA for miRNA, and the recent finding that miRNA enhances glycolysis in HBV-positive cells and promotes HCC cells’ development (Chen et al., 2021) give us a hint. LncRNA may act as ceRNA to promote antiviral innate immunity by interacting with MAVS to dissociate HK2 from mitochondria. As a pathway regulated by many lncRNAs, RIG-I-MAVS, its upregulation or downregulation may also result from downregulation or upregulation of glycolytic flux. However, these conjectures need to be explored through experimentation. With the SARS-CoV-2 pandemic, data suggest that patients with underlying conditions, including diabetes, have more severe infections and sequelae. SARS-CoV-2, as an RNA virus, shifts cell metabolism from oxidative phosphorylation to glycolysis, which triggers excessive inflammatory responses and cytokine storms (Mahrooz et al., 2021), indicating that cellular metabolism is closely linked to SARS-CoV-2 infection. LncRNA is differentially expressed in COVID-19-infected patients (Devadoss et al., 2021; Taheri et al., 2021). Therefore, the speculation above may also occur in SARS-CoV-2 infection, giving us a great impetus to explore the relationship between LncRNA, glycolysis, and antiviral innate immunity.

## Author Contributions

ZR, YY, and CC contributed equally to this work and should be considered co-first authors. All authors contributed to the article and approved the submitted version.

## Funding

This work was supported by Agricultural Industrial Technology System of Sichuan Provincial Department of Agriculture (CARS-SVDIP; “National Agricultural Industry Technology System Sichuan Veterinary Drug Innovation Team Special Project and Key”); R&D Key research and development project of Sichuan science and technology plan (2020YFN0147; “Research and integration demonstration of key technologies for improving quality and efficiency of Sichuan pig industry chain”); and Key R&D Program of Sichuan Province (2018NZ0151; “Study on comprehensive control technology of African classical swine fever”).

## Conflict of Interest

This article has not been published elsewhere in whole or in part. All authors have read and approved the content and agree to submit it for consideration for publication in the journal. All work complies with the Ethical Policies of *Frontiers in Microbiology* and has been conducted under internationally accepted ethical standards after relevant ethical review.

The authors declare that the research was conducted in the absence of any commercial or financial relationships that could be construed as a potential conflict of interest.

## Publisher’s Note

All claims expressed in this article are solely those of the authors and do not necessarily represent those of their affiliated organizations, or those of the publisher, the editors and the reviewers. Any product that may be evaluated in this article, or claim that may be made by its manufacturer, is not guaranteed or endorsed by the publisher.

## Abbreviations

PKM2: Pyruvate kinase M2
HK: hexokinase
LDHA: lactate dehydrogenase A
PFK: phosphofructokinase
RAF: tumor necrosis factor receptor-associated factors
NEMO: NF-κB essential modulator
TBK1: TANK-binding kinase 1
ISGs: IFN-stimulated genes
IRFs: interferon regulatory factors
JAK: Janus Kinase
STAT: signal transducer and activator of transcription

## References

[ref1] Ahmad I. Valverde A. Ahmad F. Naqvi A. R. (2020). Long noncoding RNA in myeloid and lymphoid cell differentiation, Polarization and Function. Cells 9:269. doi: 10.3390/cells9020269, PMID: 31979061 PMC7072530

[ref2] Assmann N. O’Brien K. L. Donnelly R. P. Dyck L. Zaiatz-Bittencourt V. Loftus R. M.. (2017). Srebp-controlled glucose metabolism is essential for NK cell functional responses. Nat. Immunol. 18, 1197–1206. doi: 10.1038/ni.3838, PMID: 28920951

[ref3] Bae S. Park P. S. U. Lee Y. Mun S. H. Giannopoulou E. Fujii T.. (2021). MYC-mediated early glycolysis negatively regulates proinflammatory responses by controlling IRF4 in inflammatory macrophages. Cell Rep. 35:109264. doi: 10.1016/j.celrep.2021.109264, PMID: 34133930 PMC8257047

[ref4] Bekkering S. Arts R. J. W. Novakovic B. Kourtzelis I. van der Heijden C. Li Y.. (2018). Metabolic induction of trained immunity through the mevalonate pathway. Cell 172, 135–146.e9. doi: 10.1016/j.cell.2017.11.025, PMID: 29328908

[ref5] Bian Z. Zhang J. Li M. Feng Y. Wang X. Zhang J.. (2018). LncRNA-FEZF1-AS1 promotes tumor proliferation and metastasis in colorectal cancer by regulating PKM2 Signaling. Clin. Cancer Res. 24, 4808–4819. doi: 10.1158/1078-0432.CCR-17-2967, PMID: 29914894

[ref6] Brand A. Singer K. Koehl G. E. Kolitzus M. Schoenhammer G. Thiel A.. (2016). LDHA-associated lactic acid production blunts tumor Immunosurveillance by T and NK cells. Cell Metab. 24, 657–671. doi: 10.1016/j.cmet.2016.08.011, PMID: 27641098

[ref7] Buck M. D. Sowell R. T. Kaech S. M. Pearce E. L. (2017). Metabolic instruction of immunity. Cell 169, 570–586. doi: 10.1016/j.cell.2017.04.004, PMID: 28475890 PMC5648021

[ref8] Buskiewicz I. A. Montgomery T. Yasewicz E. C. Huber S. A. Murphy M. P. Hartley R. C.. (2016). Reactive oxygen species induce virus-independent MAVS oligomerization in systemic lupus erythematosus. Sci. Signal. 9:ra115. doi: 10.1126/scisignal.aaf1933, PMID: 27899525 PMC5321043

[ref9] Cai C. Tang Y. D. Xu G. Zheng C. (2021). The crosstalk between viral RNA- and DNA-sensing mechanisms. Cell. Mol. Life Sci. doi: 10.1007/s00018-021-04001-7 [Epub ahead of print]. PMC8554519 34714359

[ref10] Cai X. Xu H. Chen Z. J. (2017). Prion-Like polymerization in immunity and inflammation. Cold Spring Harb. Perspect. Biol. 9:a023580. doi: 10.1101/cshperspect.a023580, PMID: 27881448 PMC5378051

[ref11] Carnero E. Barriocanal M. Prior C. Pablo Unfried J. Segura V. Guruceaga E.. (2016). Long noncoding RNA EGOT negatively affects the antiviral response and favors HCV replication. EMBO Rep. 17, 1013–1028. doi: 10.15252/embr.201541763, PMID: 27283940 PMC4931568

[ref12] Carpenter S. Aiello D. Atianand M. K. Ricci E. P. Gandhi P. Hall L. L.. (2013). A long noncoding RNA mediates both activation and repression of immune response genes. Science 341, 789–792. doi: 10.1126/science.1240925, PMID: 23907535 PMC4376668

[ref13] Chao C. C. Gutierrez-Vazquez C. Rothhammer V. Mayo L. Wheeler M. A. Tjon E. C.. (2019). Metabolic control of astrocyte pathogenic activity via cPLA2-MAVS. Cell 179, 1483–1498.e22. doi: 10.1016/j.cell.2019.11.016, PMID: 31813625 PMC6936326

[ref14] Chapman E. G. Costantino D. A. Rabe J. L. Moon S. L. Wilusz J. Nix J. C.. (2014). The STAT3-binding long noncoding RNA lnc-DC controls human dendritic cell differentiation. Science 344, 307–310. doi: 10.1126/science.1250897, PMID: 24744378

[ref15] Chauhan A. S. Zhuang L. Gan B. (2019). Antagonism between antiviral Signaling and glycolysis. Trends Endocrinol. Metab. 30, 571–573. doi: 10.1016/j.tem.2019.07.010, PMID: 31349923 PMC7944876

[ref16] Chen W. Jiang J. Gong L. Shu Z. Xiang D. Zhang X.. (2021). Hepatitis B virus P protein initiates glycolytic bypass in HBV-related hepatocellular carcinoma via a FOXO3/miRNA-30b-5p/MINPP1 axis. J. Exp. Clin. Cancer Res. 40:1. doi: 10.1186/s13046-020-01803-8, PMID: 33390177 PMC7779247

[ref17] Chen K. Xiao F. Hu D. Ge W. Tian M. Wang W.. (2020). SARS-CoV-2 Nucleocapsid protein interacts with RIG-I and represses RIG-mediated IFN-beta production. Viruses 13:47. doi: 10.3390/v13010047, PMID: 33396605 PMC7823417

[ref18] Chen J. Yu Y. Li H. Hu Q. Chen X. He Y.. (2019). Long non-coding RNA PVT1 promotes tumor progression by regulating the miR-143/HK2 axis in gallbladder cancer. Mol. Cancer 18:33. doi: 10.1186/s12943-019-0947-9, PMID: 30825877 PMC6397746

[ref19] Cheng T. Y. Lin Y. J. Saburi W. Vieths S. Scheurer S. Schulke S.. (2021b). Beta-(1-->4)-Mannobiose acts as an Immunostimulatory molecule in murine dendritic cells by binding the TLR4/MD-2 complex. Cell 10:1774. doi: 10.3390/cells10071774, PMID: 34359943 PMC8305851

[ref20] Cheng Z. Luo C. Guo Z. (2020). LncRNA-XIST/microRNA-126 sponge mediates cell proliferation and glucose metabolism through the IRS1/PI3K/Akt pathway in glioma. J. Cell. Biochem. 121, 2170–2183. doi: 10.1002/jcb.29440, PMID: 31680298

[ref21] Cheng S. C. Quintin J. Cramer R. A. Shepardson K. M. Saeed S. Kumar V.. (2014). mTOR- and HIF-1alpha-mediated aerobic glycolysis as metabolic basis for trained immunity. Science 345:1250684. doi: 10.1126/science.1250684, PMID: 25258083 PMC4226238

[ref22] Cheng S. C. Scicluna B. P. Arts R. J. Gresnigt M. S. Lachmandas E. Giamarellos-Bourboulis E. J.. (2016). Broad defects in the energy metabolism of leukocytes underlie immunoparalysis in sepsis. Nat. Immunol. 17, 406–413. doi: 10.1038/ni.3398, PMID: 26950237

[ref23] Cheng J. Zhang R. Xu Z. Ke Y. Sun R. Yang H.. (2021a). Early glycolytic reprogramming controls microglial inflammatory activation. J. Neuroinflammation 18:129. doi: 10.1186/s12974-021-02187-y, PMID: 34107997 PMC8191212

[ref24] Cheung P. H. Ye Z. W. Lee T. T. Chen H. Chan C. P. Jin D. Y. (2020). PB1-F2 protein of highly pathogenic influenza A (H7N9) virus selectively suppresses RNA-induced NLRP3 inflammasome activation through inhibition of MAVS-NLRP3 interaction. J. Leukoc. Biol. 108, 1655–1663. doi: 10.1002/JLB.4AB0420-694R, PMID: 32386456

[ref25] Chi P. I. Huang W. R. Chiu H. C. Li J. Y. Nielsen B. L. Liu H. J. (2018). Avian reovirus sigmaA-modulated suppression of lactate dehydrogenase and upregulation of glutaminolysis and the mTOC1/eIF4E/HIF-1alpha pathway to enhance glycolysis and the TCA cycle for virus replication. Cell. Microbiol. 20:e12946. doi: 10.1111/cmi.12946, PMID: 30156372

[ref26] Chu Q. Xu T. Zheng W. Chang R. Zhang L. (2020). Long noncoding RNA MARL regulates antiviral responses through suppression miR-122-dependent MAVS downregulation in lower vertebrates. PLoS Pathog. 16:e1008670. doi: 10.1371/journal.ppat.1008670, PMID: 32678830 PMC7390449

[ref27] Codo A. C. Davanzo G. G. Monteiro L. B. de Souza G. F. Muraro S. P. Virgilio-da-Silva J. V.. (2020). Elevated glucose levels favor SARS-CoV-2 infection and monocyte response through a HIF-1alpha/glycolysis-dependent Axis. Cell Metab. 32, 437–446.e5. doi: 10.1016/j.cmet.2020.07.007, PMID: 32697943 PMC7367032

[ref28] Colegio O. R. Chu N. Q. Szabo A. L. Chu T. Rhebergen A. M. Jairam V.. (2014). Functional polarization of tumour-associated macrophages by tumour-derived lactic acid. Nature 513, 559–563. doi: 10.1038/nature13490, PMID: 25043024 PMC4301845

[ref29] Condon K. J. Sabatini D. M. (2019). Nutrient regulation of mTORC1 at a glance. J. Cell Sci. 132:jcs222570. doi: 10.1242/jcs.222570, PMID: 31722960 PMC6857595

[ref30] Covian C. Fernandez-Fierro A. Retamal-Diaz A. Diaz F. E. Vasquez A. E. Lay M. K.. (2019). BCG-induced cross-protection and development of trained immunity: implication for vaccine design. Front. Immunol. 10:2806. doi: 10.3389/fimmu.2019.02806, PMID: 31849980 PMC6896902

[ref31] Cui H. Xie N. Tan Z. Banerjee S. Thannickal V. J. Abraham E.. (2014). The human long noncoding RNA lnc-IL7R regulates the inflammatory response. Eur. J. Immunol. 44, 2085–2095. doi: 10.1002/eji.201344126, PMID: 24723426 PMC4107034

[ref32] Das S. Reddy M. A. Senapati P. Stapleton K. Lanting L. Wang M.. (2018). Diabetes mellitus-induced long noncoding RNA Dnm3os regulates macrophage functions and inflammation via nuclear mechanisms. Arterioscler. Thromb. Vasc. Biol. 38, 1806–1820. doi: 10.1161/ATVBAHA.117.310663, PMID: 29930005 PMC6202204

[ref33] Devadoss D. Acharya A. Manevski M. Pandey K. Borchert G. M. Nair M.. (2021). Distinct Mucoinflammatory phenotype and the immunomodulatory long noncoding transcripts associated with SARS-CoV-2 airway infection. medRxiv [Preprint]. doi: 10.1101/2021.05.13.21257152

[ref34] DeWaal D. Nogueira V. Terry A. R. Patra K. C. Jeon S. M. Guzman G.. (2018). Hexokinase-2 depletion inhibits glycolysis and induces oxidative phosphorylation in hepatocellular carcinoma and sensitizes to metformin. Nat. Commun. 9:446. doi: 10.1038/s41467-017-02733-4, PMID: 29386513 PMC5792493

[ref35] Dong P. Xiong Y. Konno Y. Ihira K. Kobayashi N. Yue J.. (2021). Long non-coding RNA DLEU2 drives EMT and glycolysis in endometrial cancer through HK2 by competitively binding with miR-455 and by modulating the EZH2/miR-181a pathway. J. Exp. Clin. Cancer Res. 40:216. doi: 10.1186/s13046-021-02018-1, PMID: 34174908 PMC8235565

[ref36] Donnelly R. P. Loftus R. M. Keating S. E. Liou K. T. Biron C. A. Gardiner C. M.. (2014). mTORC1-dependent metabolic reprogramming is a prerequisite for NK cell effector function. J. Immunol. 193, 4477–4484. doi: 10.4049/jimmunol.1401558, PMID: 25261477 PMC4201970

[ref37] Dunn D. M. Rodriguez-Sanchez I. Schafer X. Munger J. (2020). Human cytomegalovirus induces the expression of the AMPKa2 subunit to drive glycolytic activation and support productive viral infection. J. Virol. 95, e01321–e01320. doi: 10.1128/JVI.01321-20, PMID: 33268515 PMC8092818

[ref38] Duvel K. Yecies J. L. Menon S. Raman P. Lipovsky A. I. Souza A. L.. (2010). Activation of a metabolic gene regulatory network downstream of mTOR complex 1. Mol. Cell 39, 171–183. doi: 10.1016/j.molcel.2010.06.022, PMID: 20670887 PMC2946786

[ref39] Edinger A. L. Linardic C. M. Chiang G. G. Thompson C. B. Abraham R. T. (2003). Differential effects of rapamycin on mammalian target of rapamycin signaling functions in mammalian cells. Cancer Res. 63, 8451–8460. doi: 10.1007/s00262-003-0421-8 PMID: 14679009

[ref40] Erlich J. R. To E. E. Liong S. Brooks R. Vlahos R. O’Leary J. J.. (2020). Targeting evolutionary conserved oxidative stress and Immunometabolic pathways for the treatment of respiratory infectious diseases. Antioxid. Redox Signal. 32, 993–1013. doi: 10.1089/ars.2020.8028, PMID: 32008371 PMC7426980

[ref41] Everts B. Amiel E. Huang S. C. Smith A. M. Chang C. H. Lam W. Y.. (2014). TLR-driven early glycolytic reprogramming via the kinases TBK1-IKKvarepsilon supports the anabolic demands of dendritic cell activation. Nat. Immunol. 15, 323–332. doi: 10.1038/ni.2833, PMID: 24562310 PMC4358322

[ref42] Fan J. Cheng M. Chi X. Liu X. Yang W. (2019). A human long non-coding RNA LncATV promotes virus replication Through restricting RIG-I-mediated innate immunity. Front. Immunol. 10:1711. doi: 10.3389/fimmu.2019.01711, PMID: 31379885 PMC6658999

[ref43] Fan S. Wu K. Zhao M. Yuan J. Ma S. Zhu E.. (2020). LDHB inhibition induces mitophagy and facilitates the progression of CSFV infection. Autophagy 17, 1–20. doi: 10.1080/15548627.2020.1823123 PMC8496725 32924761

[ref44] Fang P. Xiang L. Chen W. Li S. Huang S. Li J.. (2019). LncRNA GAS5 enhanced the killing effect of NK cell on liver cancer through regulating miR-544/RUNX3. Innate Immun. 25, 99–109. doi: 10.1177/1753425919827632, PMID: 30774011 PMC6830859

[ref45] Gao J. Pan Y. Xu Y. Zhang W. Zhang L. Li X.. (2021). Unveiling the long non-coding RNA profile of porcine reproductive and respiratory syndrome virus-infected porcine alveolar macrophages. BMC Genomics 22:177. doi: 10.1186/s12864-021-07482-9, PMID: 33711920 PMC7953715

[ref46] Gomez J. A. Wapinski O. L. Yang Y. W. Bureau J. F. Gopinath S. Monack D. M.. (2013). The NeST long ncRNA controls microbial susceptibility and epigenetic activation of the interferon-gamma locus. Cell 152, 743–754. doi: 10.1016/j.cell.2013.01.015, PMID: 23415224 PMC3577098

[ref47] Gowans G. J. Hawley S. A. Ross F. A. Hardie D. G. (2013). AMP is a true physiological regulator of AMP-activated protein kinase by both allosteric activation and enhancing net phosphorylation. Cell Metab. 18, 556–566. doi: 10.1016/j.cmet.2013.08.019, PMID: 24093679 PMC3791399

[ref48] Hagiwara A. Cornu M. Cybulski N. Polak P. Betz C. Trapani F.. (2012). Hepatic mTORC2 activates glycolysis and lipogenesis through Akt, glucokinase, and SREBP1c. Cell Metab. 15, 725–738. doi: 10.1016/j.cmet.2012.03.015, PMID: 22521878

[ref49] Hanada Y. Ishihara N. Wang L. Otera H. Ishihara T. Koshiba T.. (2020). MAVS is energized by Mff which senses mitochondrial metabolism via AMPK for acute antiviral immunity. Nat. Commun. 11:5711. doi: 10.1038/s41467-020-19287-7, PMID: 33177519 PMC7658986

[ref50] Hartana C. A. Rassadkina Y. Gao C. Martin-Gayo E. Walker B. D. Lichterfeld M.. (2021). Long noncoding RNA MIR4435-2HG enhances metabolic function of myeloid dendritic cells from HIV-1 elite controllers. J. Clin. Invest. 131:e146136. doi: 10.1172/JCI146136, PMID: 33938445 PMC8087208

[ref51] He L. Zhao X. He L. (2020). LINC01140 alleviates the oxidized low-density lipoprotein-induced inflammatory response in macrophages via suppressing miR-23b. Inflammation 43, 66–73. doi: 10.1007/s10753-019-01094-y, PMID: 31748847

[ref52] Hou F. Sun L. Zheng H. Skaug B. Jiang Q. X. Chen Z. J. (2011). MAVS forms functional prion-like aggregates to activate and propagate antiviral innate immune response. Cell 146, 448–461. doi: 10.1016/j.cell.2011.06.041, PMID: 21782231 PMC3179916

[ref53] Houddane A. Bultot L. Novellasdemunt L. Johanns M. Gueuning M. A. Vertommen D.. (2017). Role of Akt/PKB and PFKFB isoenzymes in the control of glycolysis, cell proliferation and protein synthesis in mitogen-stimulated thymocytes. Cell. Signal. 34, 23–37. doi: 10.1016/j.cellsig.2017.02.019, PMID: 28235572

[ref54] Hung C. L. Wang L. Y. Yu Y. L. Chen H. W. Srivastava S. Petrovics G.. (2014). A long noncoding RNA connects c-Myc to tumor metabolism. Proc. Natl. Acad. Sci. U. S. A. 111, 18697–18702. doi: 10.1073/pnas.1415669112, PMID: 25512540 PMC4284533

[ref55] Ieronymaki E. Daskalaki M. G. Lyroni K. Tsatsanis C. (2019). Insulin Signaling and insulin resistance facilitate trained immunity in macrophages Through metabolic and epigenetic changes. Front. Immunol. 10:1330. doi: 10.3389/fimmu.2019.01330, PMID: 31244863 PMC6581697

[ref56] Ivashkiv L. B. (2020). The hypoxia-lactate axis tempers inflammation. Nat. Rev. Immunol. 20, 85–86. doi: 10.1038/s41577-019-0259-8, PMID: 31819164 PMC7021227

[ref57] Jang K. J. Jeong S. Kang D. Y. Sp N. Yang Y. M. Kim D. E. (2020). A high ATP concentration enhances the cooperative translocation of the SARS coronavirus helicase nsP13 in the unwinding of duplex RNA. Sci. Rep. 10:4481. doi: 10.1038/s41598-020-61432-1, PMID: 32161317 PMC7066239

[ref58] Jia H. Liu C. Li D. Huang Q. Liu D. Zhang Y.. (2021). Metabolomic analyses reveals new stage-specific features of the COVID-19. Eur. Respir. J. doi: 10.1183/13993003.00284-2021 [Epub ahead of print]. PMC8311281 34289974

[ref59] Jiang M. Zhang S. Yang Z. Lin H. Zhu J. Liu L.. (2018). Self-recognition of an inducible host lncRNA by RIG-I feedback restricts innate immune response. Cell 173, 906–919.e13. doi: 10.1016/j.cell.2018.03.064, PMID: 29706547

[ref60] Ju X. Li F. Li J. Wu C. Xiang G. Zhao X.. (2021). Genome-wide transcriptomic analysis of highly virulent African swine fever virus infection reveals complex and unique virus host interaction. Vet. Microbiol. 261:109211. doi: 10.1016/j.vetmic.2021.109211, PMID: 34481273

[ref61] Kaidanovich-Beilin O. Woodgett J. R. (2011). GSK-3: functional insights from cell biology and animal models. Front. Mol. Neurosci. 4:40. doi: 10.3389/fnmol.2011.00040, PMID: 22110425 PMC3217193

[ref62] Keating S. T. Groh L. van der Heijden C. Rodriguez H. Dos Santos J. C. Fanucchi S.. (2020). The Set7 lysine methyltransferase regulates plasticity in oxidative phosphorylation necessary for trained immunity induced by beta-glucan. Cell Rep. 31:107548. doi: 10.1016/j.celrep.2020.107548, PMID: 32320649 PMC7184679

[ref63] Khatun M. Sur S. Steele R. Ray R. Ray R. B. (2021). Inhibition of long noncoding RNA Linc-pint by hepatitis C virus in infected hepatocytes enhances lipogenesis. Hepatology 74, 41–54. doi: 10.1002/hep.31656, PMID: 33236406 PMC8141542

[ref64] Kim J. (2018). Regulation of immune cell functions by metabolic reprogramming. J Immunol Res 2018:8605471. doi: 10.1155/2018/8605471, PMID: 29651445 PMC5831954

[ref65] Kim J. W. Tchernyshyov I. Semenza G. L. Dang C. V. (2006). HIF-1-mediated expression of pyruvate dehydrogenase kinase: a metabolic switch required for cellular adaptation to hypoxia. Cell Metab. 3, 177–185. doi: 10.1016/j.cmet.2006.02.002, PMID: 16517405

[ref66] Kohio H. P. Adamson A. L. (2013). Glycolytic control of vacuolar-type ATPase activity: a mechanism to regulate influenza viral infection. Virology 444, 301–309. doi: 10.1016/j.virol.2013.06.026, PMID: 23876457

[ref67] Krawczyk C. M. Holowka T. Sun J. Blagih J. Amiel E. DeBerardinis R. J.. (2010). Toll-like receptor-induced changes in glycolytic metabolism regulate dendritic cell activation. Blood 115, 4742–4749. doi: 10.1182/blood-2009-10-249540, PMID: 20351312 PMC2890190

[ref68] Kumar H. Kawai T. Kato H. Sato S. Takahashi K. Coban C.. (2006). Essential role of IPS-1 in innate immune responses against RNA viruses. J. Exp. Med. 203, 1795–1803. doi: 10.1084/jem.20060792, PMID: 16785313 PMC2118350

[ref69] Lai C. Liu L. Liu Q. Wang K. Cheng S. Zhao L.. (2021). Long noncoding RNA AVAN promotes antiviral innate immunity by interacting with TRIM25 and enhancing the transcription of FOXO3a. Cell Death Differ. 28, 2900–2915. doi: 10.1038/s41418-021-00791-2, PMID: 33990776 PMC8481484

[ref70] Lauer V. Grampp S. Platt J. Lafleur V. Lombardi O. Choudhry H.. (2020). Hypoxia drives glucose transporter 3 expression through hypoxia-inducible transcription factor (HIF)-mediated induction of the long noncoding RNA NICI. J. Biol. Chem. 295, 4065–4078. doi: 10.1074/jbc.RA119.009827, PMID: 31690629 PMC7105321

[ref71] Li X. Guo G. Lu M. Chai W. Li Y. Tong X.. (2019). Long noncoding RNA Lnc-MxA inhibits Beta interferon transcription by forming RNA-DNA triplexes at its promoter. J. Virol. 93, e00786–e00719. doi: 10.1128/JVI.00786-19, PMID: 31434735 PMC6803265

[ref72] Li X. Liu R. Wang Y. Zhu W. Zhao D. Wang X.. (2020). Cholangiocyte-derived Exosomal lncRNA H19 promotes macrophage activation and hepatic inflammation under Cholestatic conditions. Cell 9:190. doi: 10.3390/cells9010190, PMID: 31940841 PMC7016679

[ref73] Lim S. A. Moon Y. Shin M. H. Kim T. J. Chae S. Yee C.. (2021). Hypoxia-driven HIF-1alpha activation reprograms pre-activated NK cells towards highly potent effector phenotypes via ERK/STAT3 pathways. Cancers 13:1904. doi: 10.3390/cancers13081904, PMID: 33920906 PMC8071270

[ref74] Lin S. C. Hardie D. G. (2018). AMPK: sensing glucose as well as cellular energy status. Cell Metab. 27, 299–313. doi: 10.1016/j.cmet.2017.10.009, PMID: 29153408

[ref75] Lin H. Jiang M. Liu L. Yang Z. Ma Z. Liu S.. (2019). The long noncoding RNA Lnczc3h7a promotes a TRIM25-mediated RIG-I antiviral innate immune response. Nat. Immunol. 20, 812–823. doi: 10.1038/s41590-019-0379-0, PMID: 31036902

[ref76] Lin Y. H. Wu M. H. Huang Y. H. Yeh C. T. Cheng M. L. Chi H. C.. (2018). Taurine up-regulated gene 1 functions as a master regulator to coordinate glycolysis and metastasis in hepatocellular carcinoma. Hepatology 67, 188–203. doi: 10.1002/hep.29462, PMID: 28802060

[ref77] Liu X. Gan B. (2016). lncRNA NBR2 modulates cancer cell sensitivity to phenformin through GLUT1. Cell Cycle 15, 3471–3481. doi: 10.1080/15384101.2016.1249545, PMID: 27792451 PMC5224451

[ref78] Liu Y. Qin C. Rao Y. Ngo C. Feng J. J. Zhao J.. (2021b). SARS-CoV-2 Nsp5 demonstrates two distinct mechanisms targeting RIG-I and MAVS To evade the innate immune response. MBio 12:e0233521. doi: 10.1128/mBio.02335-21, PMID: 34544279 PMC8546575

[ref79] Liu D. Tan Q. Zhu J. Zhang Y. Xue Y. Song Y.. (2021a). MicroRNA-33/33* inhibit the activation of MAVS through AMPK in antiviral innate immunity. Cell. Mol. Immunol. 18, 1450–1462. doi: 10.1038/s41423-019-0326-x, PMID: 31767975 PMC8167167

[ref80] Liu J. Wang F. Du L. Li J. Yu T. Jin Y.. (2019a). Comprehensive genomic characterization analysis of lncRNAs in cells With Porcine Delta coronavirus infection. Front. Microbiol. 10:3036. doi: 10.3389/fmicb.2019.03036, PMID: 32063887 PMC6999024

[ref81] Liu J. Zhang X. Chen K. Cheng Y. Liu S. Xia M.. (2019b). CCR7 chemokine receptor-inducible lnc-Dpf3 restrains dendritic cell migration by inhibiting HIF-1alpha-mediated glycolysis. Immunity 50, 600–615.e15. doi: 10.1016/j.immuni.2019.01.021, PMID: 30824325

[ref82] Liu Z. Zhao P. Han Y. Lu S. (2018). lncRNA FEZF1-AS1 is associated With prognosis in lung adenocarcinoma and promotes cell proliferation, migration, and invasion. Oncol. Res. 27, 39–45. doi: 10.3727/096504018X15199482824130, PMID: 29510777 PMC7848278

[ref83] Locasale J. W. Cantley L. C. (2011). Metabolic flux and the regulation of mammalian cell growth. Cell Metab. 14, 443–451. doi: 10.1016/j.cmet.2011.07.014, PMID: 21982705 PMC3196640

[ref84] Loftus R. M. Assmann N. Kedia-Mehta N. O’Brien K. L. Garcia A. Gillespie C.. (2018). Amino acid-dependent cMyc expression is essential for NK cell metabolic and functional responses in mice. Nat. Commun. 9:2341. doi: 10.1038/s41467-018-04719-2, PMID: 29904050 PMC6002377

[ref85] Luo J. Wang H. Wang L. Wang G. Yao Y. Xie K.. (2021). lncRNA GAS6-AS1 inhibits progression and glucose metabolism reprogramming in LUAD via repressing E2F1-mediated transcription of GLUT1. Mol. Ther. Nucleic Acids 25, 11–24. doi: 10.1016/j.omtn.2021.04.022, PMID: 34141461 PMC8181633

[ref86] Mah A. Y. Rashidi A. Keppel M. P. Saucier N. Moore E. K. Alinger J. B.. (2017). Glycolytic requirement for NK cell cytotoxicity and cytomegalovirus control. JCI Insight 2:e95128. doi: 10.1172/jci.insight.95128, PMID: 29212951 PMC5752285

[ref87] Mahrooz A. Muscogiuri G. Buzzetti R. Maddaloni E. (2021). The complex combination of COVID-19 and diabetes: pleiotropic changes in glucose metabolism. Endocrine 72, 317–325. doi: 10.1007/s12020-021-02729-7, PMID: 33886062 PMC8060688

[ref88] Mansouri K. Rastegari-Pouyani M. Ghanbri-Movahed M. Safarzadeh M. Kiani S. Ghanbari-Movahed Z. (2020). Can a metabolism-targeted therapeutic intervention successfully subjugate SARS-COV-2? Biomed. Pharmacother. 131:110694. doi: 10.1016/j.biopha.2020.110694, PMID: 32920511 PMC7451059

[ref89] Marcais A. Cherfils-Vicini J. Viant C. Degouve S. Viel S. Fenis A.. (2014). The metabolic checkpoint kinase mTOR is essential for IL-15 signaling during the development and activation of NK cells. Nat. Immunol. 15, 749–757. doi: 10.1038/ni.2936, PMID: 24973821 PMC4110708

[ref90] Marques A. C. Ponting C. P. (2009). Catalogues of mammalian long noncoding RNAs: modest conservation and incompleteness. Genome Biol. 10:R124. doi: 10.1186/gb-2009-10-11-r124, PMID: 19895688 PMC3091318

[ref91] McElvaney O. J. McEvoy N. L. McElvaney O. F. Carroll T. P. Murphy M. P. Dunlea D. M.. (2020). Characterization of the inflammatory response to severe COVID-19 illness. Am. J. Respir. Crit. Care Med. 202, 812–821. doi: 10.1164/rccm.202005-1583OC, PMID: 32584597 PMC7491404

[ref92] Mills E. L. Kelly B. O’Neill L. A. J. (2017). Mitochondria are the powerhouses of immunity. Nat. Immunol. 18, 488–498. doi: 10.1038/ni.3704, PMID: 28418387

[ref93] Moon J. S. Hisata S. Park M. A. DeNicola G. M. Ryter S. W. Nakahira K.. (2015). mTORC1-induced HK1-dependent glycolysis regulates NLRP3 Inflammasome activation. Cell Rep. 12, 102–115. doi: 10.1016/j.celrep.2015.05.046, PMID: 26119735 PMC4858438

[ref94] Netea M. G. Quintin J. van der Meer J. W. (2011). Trained immunity: a memory for innate host defense. Cell Host Microbe 9, 355–361. doi: 10.1016/j.chom.2011.04.006, PMID: 21575907

[ref95] Nguyen M. H. Wong G. Gane E. Kao J. H. Dusheiko G. (2020). Hepatitis B virus: advances in prevention, diagnosis, and therapy. Clin. Microbiol. Rev. 33, e00046–e00019. doi: 10.1128/CMR.00046-19, PMID: 32102898 PMC7048015

[ref96] Nie J. Zhao Q. (2020). Lnc-ITSN1-2, derived From RNA sequencing, correlates With increased disease risk, activity and promotes CD4(+) T cell activation, proliferation and Th1/Th17 cell differentiation by serving as a ceRNA for IL-23R via sponging miR-125a in inflammatory bowel disease. Front. Immunol. 11:852. doi: 10.3389/fimmu.2020.00852, PMID: 32547537 PMC7271921

[ref97] Nomura N. Verdon G. Kang H. J. Shimamura T. Nomura Y. Sonoda Y.. (2015). Structure and mechanism of the mammalian fructose transporter GLUT5. Nature 526, 397–401. doi: 10.1038/nature14909, PMID: 26416735 PMC4618315

[ref98] O’Brien K. L. Finlay D. K. (2019). Immunometabolism and natural killer cell responses. Nat. Rev. Immunol. 19, 282–290. doi: 10.1038/s41577-019-0139-2, PMID: 30808985

[ref99] O’Neill L. A. Hardie D. G. (2013). Metabolism of inflammation limited by AMPK and pseudo-starvation. Nature 493, 346–355. doi: 10.1038/nature11862, PMID: 23325217

[ref100] O’Neill L. A. Kishton R. J. Rathmell J. (2016). A guide to immunometabolism for immunologists. Nat. Rev. Immunol. 16, 553–565. doi: 10.1038/nri.2016.70, PMID: 27396447 PMC5001910

[ref101] Oshiumi H. Miyashita M. Inoue N. Okabe M. Matsumoto M. Seya T. (2010). The ubiquitin ligase Riplet is essential for RIG-I-dependent innate immune responses to RNA virus infection. Cell Host Microbe 8, 496–509. doi: 10.1016/j.chom.2010.11.008, PMID: 21147464

[ref102] Osthus R. C. Shim H. Kim S. Li Q. Reddy R. Mukherjee M.. (2000). Deregulation of glucose transporter 1 and glycolytic gene expression by c-Myc. J. Biol. Chem. 275, 21797–21800. doi: 10.1074/jbc.C000023200, PMID: 10823814

[ref103] Ouyang J. Zhu X. Chen Y. Wei H. Chen Q. Chi X.. (2014). NRAV, a long noncoding RNA, modulates antiviral responses through suppression of interferon-stimulated gene transcription. Cell Host Microbe 16, 616–626. doi: 10.1016/j.chom.2014.10.001, PMID: 25525793 PMC7104942

[ref104] Palsson-McDermott E. M. Curtis A. M. Goel G. Lauterbach M. A. Sheedy F. J. Gleeson L. E.. (2015). Pyruvate kinase M2 regulates Hif-1alpha activity and IL-1beta induction and is a critical determinant of the Warburg effect in LPS-activated macrophages. Cell Metab. 21, 65–80. doi: 10.1016/j.cmet.2014.12.005, PMID: 25565206 PMC5198835

[ref105] Pan Q. Zhao Z. Liao Y. Chiu S. H. Wang S. Chen B.. (2019). Identification of an interferon-stimulated long noncoding RNA (LncRNA ISR) involved in regulation of influenza A virus replication. Int. J. Mol. Sci. 20:5118. doi: 10.3390/ijms20205118, PMID: 31623059 PMC6829313

[ref106] Passalacqua K. D. Lu J. Goodfellow I. Kolawole A. O. Arche J. R. Maddox R. J.. (2019). Glycolysis is an intrinsic factor for optimal replication of a norovirus. MBio 10, e02175–e02118. doi: 10.1128/mBio.02175-18, PMID: 30862747 PMC6414699

[ref107] Pearce E. L. Poffenberger M. C. Chang C. H. Jones R. G. (2013). Fueling immunity: insights into metabolism and lymphocyte function. Science 342:1242454. doi: 10.1126/science.1242454, PMID: 24115444 PMC4486656

[ref108] Peng M. Yin N. Chhangawala S. Xu K. Leslie C. S. Li M. O. (2016). Aerobic glycolysis promotes T helper 1 cell differentiation through an epigenetic mechanism. Science 354, 481–484. doi: 10.1126/science.aaf6284, PMID: 27708054 PMC5539971

[ref109] Prantner D. Perkins D. J. Vogel S. N. (2017). AMP-activated kinase (AMPK) promotes innate immunity and antiviral Defense through modulation of stimulator of interferon genes (STING) Signaling. J. Biol. Chem. 292, 292–304. doi: 10.1074/jbc.M116.763268, PMID: 27879319 PMC5217687

[ref110] Prusinkiewicz M. A. Mymryk J. S. (2021). Metabolic control by DNA tumor virus-encoded proteins. Pathogens 10:560. doi: 10.3390/pathogens10050560, PMID: 34066504 PMC8148605

[ref111] Ramiere C. Rodriguez J. Enache L. S. Lotteau V. Andre P. Diaz O. (2014). Activity of hexokinase is increased by its interaction with hepatitis C virus protein NS5A. J. Virol. 88, 3246–3254. doi: 10.1128/JVI.02862-13, PMID: 24390321 PMC3957934

[ref112] Rapicavoli N. A. Qu K. Zhang J. Mikhail M. Laberge R. M. Chang H. Y. (2013). A mammalian pseudogene lncRNA at the interface of inflammation and anti-inflammatory therapeutics. elife 2:e00762. doi: 10.7554/eLife.00762, PMID: 23898399 PMC3721235

[ref113] Ren Z. Ding T. Zuo Z. Xu Z. Deng J. Wei Z. (2020). Regulation of MAVS expression and Signaling function in the antiviral innate immune response. Front. Immunol. 11:1030. doi: 10.3389/fimmu.2020.01030, PMID: 32536927 PMC7267026

[ref114] Rezinciuc S. Bezavada L. Bahadoran A. Duan S. Wang R. Lopez-Ferrer D.. (2020). Dynamic metabolic reprogramming in dendritic cells: An early response to influenza infection that is essential for effector function. PLoS Pathog. 16:e1008957. doi: 10.1371/journal.ppat.1008957, PMID: 33104753 PMC7707590

[ref115] Riksen N. P. Netea M. G. (2021). Immunometabolic control of trained immunity. Mol. Asp. Med. 77:100897. doi: 10.1016/j.mam.2020.100897, PMID: 32891423 PMC7466946

[ref116] Ripoli M. D’Aprile A. Quarato G. Sarasin-Filipowicz M. Gouttenoire J. Scrima R.. (2010). Hepatitis C virus-linked mitochondrial dysfunction promotes hypoxia-inducible factor 1 alpha-mediated glycolytic adaptation. J. Virol. 84, 647–660. doi: 10.1128/JVI.00769-09, PMID: 19846525 PMC2798449

[ref117] Roberts D. J. Miyamoto S. (2015). Hexokinase II integrates energy metabolism and cellular protection: Akting on mitochondria and TORCing to autophagy. Cell Death Differ. 22, 248–257. doi: 10.1038/cdd.2014.173, PMID: 25323588 PMC4291497

[ref118] Roberts D. J. Tan-Sah V. P. Smith J. M. Miyamoto S. (2013). Akt phosphorylates HK-II at Thr-473 and increases mitochondrial HK-II association to protect cardiomyocytes. J. Biol. Chem. 288, 23798–23806. doi: 10.1074/jbc.M113.482026, PMID: 23836898 PMC3745326

[ref119] Ryu W. I. Bormann M. K. Shen M. Kim D. Forester B. Park Y.. (2021). Brain cells derived from Alzheimer’s disease patients have multiple specific innate abnormalities in energy metabolism. Mol. Psychiatry doi: 10.1038/s41380-021-01068-3 [Epub ahead of print]. PMC8758493 33863993

[ref120] Sanchez-Aparicio M. T. Ayllon J. Leo-Macias A. Wolff T. Garcia-Sastre A. (2017). Subcellular localizations of RIG-I, TRIM25, and MAVS complexes. J. Virol. 91, e01155–e01116. doi: 10.1128/JVI.01155-16, PMID: 27807226 PMC5215348

[ref121] Schlee M. Hartmann G. (2016). Discriminating self from non-self in nucleic acid sensing. Nat. Rev. Immunol. 16, 566–580. doi: 10.1038/nri.2016.78, PMID: 27455396 PMC7097691

[ref122] Seagroves T. N. Ryan H. E. Lu H. Wouters B. G. Knapp M. Thibault P.. (2001). Transcription factor HIF-1 is a necessary mediator of the pasteur effect in mammalian cells. Mol. Cell. Biol. 21, 3436–3444. doi: 10.1128/MCB.21.10.3436-3444.2001, PMID: 11313469 PMC100265

[ref123] Seth R. B. Sun L. Ea C. K. Chen Z. J. (2005). Identification and characterization of MAVS, a mitochondrial antiviral signaling protein that activates NF-kappaB and IRF 3. Cell 122, 669–682. doi: 10.1016/j.cell.2005.08.012, PMID: 16125763

[ref124] Shang R. Wang M. Dai B. Du J. Wang J. Liu Z.. (2020). Long noncoding RNA SLC2A1-AS1 regulates aerobic glycolysis and progression in hepatocellular carcinoma via inhibiting the STAT3/FOXM1/GLUT1 pathway. Mol. Oncol. 14, 1381–1396. doi: 10.1002/1878-0261.12666, PMID: 32174012 PMC7266282

[ref125] Sharma G. Tripathi S. K. Das S. (2019). lncRNA HULC facilitates efficient loading of HCV-core protein onto lipid droplets and subsequent virus-particle release. Cell. Microbiol. 21:e13086. doi: 10.1111/cmi.13086, PMID: 31290220

[ref126] Sheppard S. Santosa E. K. Lau C. M. Violante S. Giovanelli P. Kim H.. (2021). Lactate dehydrogenase A-dependent aerobic glycolysis promotes natural killer cell anti-viral and anti-tumor function. Cell Rep. 35:109210. doi: 10.1016/j.celrep.2021.109210, PMID: 34077737 PMC8221253

[ref127] Shirahama S. Onoguchi-Mizutani R. Kawata K. Taniue K. Miki A. Kato A.. (2020). Long noncoding RNA U90926 is crucial for herpes simplex virus type 1 proliferation in murine retinal photoreceptor cells. Sci. Rep. 10:19406. doi: 10.1038/s41598-020-76450-2, PMID: 33173149 PMC7656448

[ref128] Silwal P. Kim J. K. Jeon S. M. Lee J. Y. Kim Y. J. Kim Y. S.. (2021). Mitofusin-2 boosts innate immunity through the maintenance of aerobic glycolysis and activation of xenophagy in mice. Commun. Biol. 4:548. doi: 10.1038/s42003-021-02073-6, PMID: 33972668 PMC8110749

[ref129] Singh S. Singh P. K. Suhail H. Arumugaswami V. Pellett P. E. Giri S.. (2020). AMP-activated protein kinase restricts Zika virus replication in endothelial cells by potentiating innate antiviral responses and inhibiting glycolysis. J. Immunol. 204, 1810–1824. doi: 10.4049/jimmunol.1901310, PMID: 32086387 PMC7310572

[ref130] Smallwood H. S. Duan S. Morfouace M. Rezinciuc S. Shulkin B. L. Shelat A.. (2017). Targeting metabolic reprogramming by influenza infection for therapeutic intervention. Cell Rep. 19, 1640–1653. doi: 10.1016/j.celrep.2017.04.039, PMID: 28538182 PMC5599215

[ref131] Smeele K. M. Southworth R. Wu R. Xie C. Nederlof R. Warley A.. (2011). Disruption of hexokinase II-mitochondrial binding blocks ischemic preconditioning and causes rapid cardiac necrosis. Circ. Res. 108, 1165–1169. doi: 10.1161/CIRCRESAHA.111.244962, PMID: 21527739

[ref132] Sohrabi Y. Lagache S. M. M. Schnack L. Godfrey R. Kahles F. Bruemmer D.. (2018). mTOR-dependent oxidative stress regulates oxLDL-induced trained innate immunity in human monocytes. Front. Immunol. 9:3155. doi: 10.3389/fimmu.2018.03155, PMID: 30723479 PMC6350618

[ref133] Stark G. R. Darnell J. E. Jr. (2012). The JAK-STAT pathway at twenty. Immunity 36, 503–514. doi: 10.1016/j.immuni.2012.03.013, PMID: 22520844 PMC3909993

[ref134] Subramanian N. Natarajan K. Clatworthy M. R. Wang Z. Germain R. N. (2013). The adaptor MAVS promotes NLRP3 mitochondrial localization and inflammasome activation. Cell 153, 348–361. doi: 10.1016/j.cell.2013.02.054, PMID: 23582325 PMC3632354

[ref135] Sun Q. Sun L. Liu H. H. Chen X. Seth R. B. Forman J.. (2006). The specific and essential role of MAVS in antiviral innate immune responses. Immunity 24, 633–642. doi: 10.1016/j.immuni.2006.04.004, PMID: 16713980

[ref136] Taheri M. Rad L. M. Hussen B. M. Nicknafs F. Sayad A. Ghafouri-Fard S. (2021). Evaluation of expression of VDR-associated lncRNAs in COVID-19 patients. BMC Infect. Dis. 21:588. doi: 10.1186/s12879-021-06248-8, PMID: 34147082 PMC8214050

[ref137] Takeuchi O. Akira S. (2010). Pattern recognition receptors and inflammation. Cell 140, 805–820. doi: 10.1016/j.cell.2010.01.022, PMID: 20303872

[ref138] Tamada M. Suematsu M. Saya H. (2012). Pyruvate kinase M2: multiple faces for conferring benefits on cancer cells. Clin. Cancer Res. 18, 5554–5561. doi: 10.1158/1078-0432.CCR-12-0859, PMID: 23071357

[ref139] Tannahill G. M. Curtis A. M. Adamik J. Palsson-McDermott E. M. McGettrick A. F. Goel G.. (2013). Succinate is an inflammatory signal that induces IL-1beta through HIF-1alpha. Nature 496, 238–242. doi: 10.1038/nature11986, PMID: 23535595 PMC4031686

[ref140] Thai M. Graham N. A. Braas D. Nehil M. Komisopoulou E. Kurdistani S. K.. (2014). Adenovirus E4ORF1-induced MYC activation promotes host cell anabolic glucose metabolism and virus replication. Cell Metab. 19, 694–701. doi: 10.1016/j.cmet.2014.03.009, PMID: 24703700 PMC4294542

[ref141] Viel S. Marcais A. Guimaraes F. S. Loftus R. Rabilloud J. Grau M.. (2016). TGF-beta inhibits the activation and functions of NK cells by repressing the mTOR pathway. Sci. Signal. 9:ra19. doi: 10.1126/scisignal.aad1884, PMID: 26884601

[ref142] Virga F. Cappellesso F. Stijlemans B. Henze A. T. Trotta R. Van Audenaerde J.. (2021). Macrophage miR-210 induction and metabolic reprogramming in response to pathogen interaction boost life-threatening inflammation. Sci. Adv. 7:eabf0466. doi: 10.1126/sciadv.abf0466, PMID: 33962944 PMC7616432

[ref143] Vivier E. Tomasello E. Baratin M. Walzer T. Ugolini S. (2008). Functions of natural killer cells. Nat. Immunol. 9, 503–510. doi: 10.1038/ni1582, PMID: 18425107

[ref144] Wang Q. Fang P. He R. Li M. Yu H. Zhou L.. (2020b). O-GlcNAc transferase promotes influenza A virus-induced cytokine storm by targeting interferon regulatory factor-5. Sci. Adv. 6:eaaz7086. doi: 10.1126/sciadv.aaz7086, PMID: 32494619 PMC7159909

[ref145] Wang Z. Guan D. Huo J. Biswas S. K. Huang Y. Yang Y.. (2021b). IL-10 enhances human natural killer cell effector functions via metabolic reprogramming regulated by mTORC1 Signaling. Front. Immunol. 12:619195. doi: 10.3389/fimmu.2021.619195, PMID: 33708210 PMC7940510

[ref146] Wang H. Liu Y. Huan C. Yang J. Li Z. Zheng B.. (2020a). Long noncoding RNA NKILA regulates HIV-1 replication and latency through repressing NF-ĸB signaling. J. Virol. 94, doi: 10.1128/JVI.01057-20 PMC7431781 32581100

[ref147] Wang Y. Wang P. Zhang Y. Xu J. Li Z. Li Z.. (2020c). Decreased expression of the host long-noncoding RNA-GM facilitates viral escape by inhibiting the kinase activity TBK1 via S-glutathionylation. Immunity 53, 1168–1181.e1167. doi: 10.1016/j.immuni.2020.11.010, PMID: 33326766

[ref148] Wang L. Xia J. W. Ke Z. P. Zhang B. H. (2019b). Blockade of NEAT1 represses inflammation response and lipid uptake via modulating miR-342-3p in human macrophages THP-1 cells. J. Cell. Physiol. 234, 5319–5326. doi: 10.1002/jcp.27340, PMID: 30259979

[ref149] Wang P. Xu J. Wang Y. Cao X. (2017). An interferon-independent lncRNA promotes viral replication by modulating cellular metabolism. Science 358, 1051–1055. doi: 10.1126/science.aao0409, PMID: 29074580

[ref150] Wang J. Yang C. Hou X. Xu J. Yun Y. Qin L.. (2021a). Rapamycin modulates the Proinflammatory memory-Like response of microglia induced by BAFF. Front. Immunol. 12:639049. doi: 10.3389/fimmu.2021.639049, PMID: 34054807 PMC8158300

[ref151] Wang J. Zhang Y. Li Q. Zhao J. Yi D. Ding J.. (2019a). Influenza virus exploits an interferon-independent lncRNA to preserve viral RNA synthesis through stabilizing viral RNA polymerase PB1. Cell Rep. 27, 3295–3304.e4. doi: 10.1016/j.celrep.2019.05.036, PMID: 31189112

[ref152] Wei S. Fan Q. Yang L. Zhang X. Ma Y. Zong Z.. (2017). Promotion of glycolysis by HOTAIR through GLUT1 upregulation via mTOR signaling. Oncol. Rep. 38, 1902–1908. doi: 10.3892/or.2017.5840, PMID: 28731193

[ref153] Wilk R. Hu J. Blotsky D. Krause H. M. (2016). Diverse and pervasive subcellular distributions for both coding and long noncoding RNAs. Genes Dev. 30, 594–609. doi: 10.1101/gad.276931.115, PMID: 26944682 PMC4782052

[ref154] Wilson J. E. (2003). Isozymes of mammalian hexokinase: structure, subcellular localization and metabolic function. J. Exp. Biol. 206, 2049–2057. doi: 10.1242/jeb.00241, PMID: 12756287

[ref155] Wolf A. J. Reyes C. N. Liang W. Becker C. Shimada K. Wheeler M. L.. (2016). Hexokinase is an innate immune receptor for the detection of bacterial peptidoglycan. Cell 166, 624–636. doi: 10.1016/j.cell.2016.05.076, PMID: 27374331 PMC5534359

[ref156] Wu Y. H. Yang Y. Chen C. H. Hsiao C. J. Li T. N. Liao K. J.. (2021). Aerobic glycolysis supports hepatitis B virus protein synthesis through interaction between viral surface antigen and pyruvate kinase isoform M2. PLoS Pathog. 17:e1008866. doi: 10.1371/journal.ppat.1008866, PMID: 33720996 PMC8009439

[ref157] Wullschleger S. Loewith R. Hall M. N. (2006). TOR signaling in growth and metabolism. Cell 124, 471–484. doi: 10.1016/j.cell.2006.01.016, PMID: 16469695

[ref158] Xiao M. Chen Y. Wang S. Liu S. Rai K. R. Chen B.. (2021). LncRNA IFITM4P regulates host antiviral responses by acting as a ceRNA. J. Virol. 95:JVI0027721. doi: 10.1128/JVI.00277-21, PMID: 34287042 PMC8513474

[ref159] Xiao Z. D. Han L. Lee H. Zhuang L. Zhang Y. Baddour J.. (2017). Energy stress-induced lncRNA FILNC1 represses c-Myc-mediated energy metabolism and inhibits renal tumor development. Nat. Commun. 8:783. doi: 10.1038/s41467-017-00902-z, PMID: 28978906 PMC5627275

[ref160] Xiao L. Hu Z. Y. Dong X. Tan Z. Li W. Tang M.. (2014). Targeting Epstein-Barr virus oncoprotein LMP1-mediated glycolysis sensitizes nasopharyngeal carcinoma to radiation therapy. Oncogene 33, 4568–4578. doi: 10.1038/onc.2014.32, PMID: 24662831 PMC4162460

[ref161] Xing J. Wang S. Lin R. Mossman K. L. Zheng C. (2012). Herpes simplex virus 1 tegument protein US11 downmodulates the RLR signaling pathway via direct interaction with RIG-I and MDA-5. J. Virol. 86, 3528–3540. doi: 10.1128/JVI.06713-11, PMID: 22301138 PMC3302539

[ref162] Xing J. Weng L. Yuan B. Wang Z. Jia L. Jin R.. (2016). Identification of a role for TRIM29 in the control of innate immunity in the respiratory tract. Nat. Immunol. 17, 1373–1380. doi: 10.1038/ni.3580, PMID: 27695001 PMC5558830

[ref163] Xing J. Zhang A. Minze L. J. Li X. C. Zhang Z. (2018). TRIM29 negatively regulates the type I IFN production in response to RNA virus. J. Immunol. 201, 183–192. doi: 10.4049/jimmunol.1701569, PMID: 29769269 PMC6092021

[ref164] Xu X. Ye L. Araki K. Ahmed R. (2012). mTOR, linking metabolism and immunity. Semin. Immunol. 24, 429–435. doi: 10.1016/j.smim.2012.12.005, PMID: 23352227 PMC3582734

[ref165] Xue G. Zippelius A. Wicki A. Mandala M. Tang F. Massi D.. (2015). Integrated Akt/PKB signaling in immunomodulation and its potential role in cancer immunotherapy. J. Natl. Cancer Inst. 107:djv171. doi: 10.1093/jnci/djv171, PMID: 26071042

[ref166] Yang W. Lu Z. (2013). Nuclear PKM2 regulates the Warburg effect. Cell Cycle 12, 3154–3158. doi: 10.4161/cc.26182, PMID: 24013426 PMC3865010

[ref167] Yang B. Zhang L. Cao Y. Chen S. Cao J. Wu D.. (2017). Overexpression of lncRNA IGFBP4-1 reprograms energy metabolism to promote lung cancer progression. Mol. Cancer 16:154. doi: 10.1186/s12943-017-0722-8, PMID: 28946875 PMC5613386

[ref168] Yu T. Yang Q. Tian F. Chang H. Hu Z. Yu B.. (2021). Glycometabolism regulates hepatitis C virus release. PLoS Pathog. 17:e1009746. doi: 10.1371/journal.ppat.1009746, PMID: 34297778 PMC8301660

[ref169] Zhang Q. Chao T. C. Patil V. S. Qin Y. Tiwari S. K. Chiou J.. (2019a). The long noncoding RNA ROCKI regulates inflammatory gene expression. EMBO J. 38:e100041. doi: 10.15252/embj.2018100041, PMID: 30918008 PMC6463213

[ref170] Zhang R. Ni F. Fu B. Wu Y. Sun R. Tian Z.. (2016). A long noncoding RNA positively regulates CD56 in human natural killer cells. Oncotarget 7, 72546–72558. doi: 10.18632/oncotarget.12466, PMID: 27713137 PMC5341928

[ref171] Zhang W. Wang G. Xu Z. G. Tu H. Hu F. Dai J.. (2019b). Lactate is a natural suppressor of RLR Signaling by targeting MAVS. Cell 178, 176–189.e15. doi: 10.1016/j.cell.2019.05.003, PMID: 31155231 PMC6625351

[ref172] Zhang H. Xue C. Wang Y. Shi J. Zhang X. Li W.. (2017). Deep RNA sequencing uncovers a repertoire of human macrophage long intergenic noncoding RNAs modulated by macrophage activation and associated With Cardiometabolic diseases. J. Am. Heart Assoc. 6:e007431. doi: 10.1161/JAHA.117.007431, PMID: 29133519 PMC5721798

[ref173] Zhao L. Xia M. Wang K. Lai C. Fan H. Gu H.. (2020). A long non-coding RNA IVRPIE promotes host antiviral immune responses Through regulating interferon beta1 and ISG expression. Front. Microbiol. 11:260. doi: 10.3389/fmicb.2020.00260, PMID: 32153544 PMC7044153

[ref174] Zheng F. Chen J. Zhang X. Wang Z. Chen J. Lin X.. (2021). The HIF-1alpha antisense long non-coding RNA drives a positive feedback loop of HIF-1alpha mediated transactivation and glycolysis. Nat. Commun. 12:1341. doi: 10.1038/s41467-021-21535-3, PMID: 33637716 PMC7910558

[ref175] Zheng H. Y. Xu M. Yang C. X. Tian R. R. Zhang M. Li J. J.. (2020). Longitudinal transcriptome analyses show robust T cell immunity during recovery from COVID-19. Signal Transduct. Target. Ther. 5:294. doi: 10.1038/s41392-020-00457-4, PMID: 33361761 PMC7758413

[ref176] Zhou L. He R. Fang P. Li M. Yu H. Wang Q.. (2021). Hepatitis B virus rigs the cellular metabolome to avoid innate immune recognition. Nat. Commun. 12:98. doi: 10.1038/s41467-020-20316-8, PMID: 33397935 PMC7782485

[ref177] Zhou R. Yazdi A. S. Menu P. Tschopp J. (2011). A role for mitochondria in NLRP3 inflammasome activation. Nature 469, 221–225. doi: 10.1038/nature09663, PMID: 21124315

[ref178] Zhu M. Cai Y. Zhao W. He C. Yang Y. Gao Q.. (2020). Long non-coding RNAs are associated with Seneca Valley virus infection. Vet. Microbiol. 246:108728. doi: 10.1016/j.vetmic.2020.108728, PMID: 32605750

[ref179] Zhu B. Wu Y. Huang S. Zhang R. Son Y. M. Li C.. (2021a). Uncoupling of macrophage inflammation from self-renewal modulates host recovery from respiratory viral infection. Immunity 54, 1200–1218.e1209. doi: 10.1016/j.immuni.2021.04.001, PMID: 33951416 PMC8192557

[ref180] Zhu L. L. Wu Z. Li R. K. Xing X. Jiang Y. S. Li J.. (2021b). Deciphering the genomic and lncRNA landscapes of aerobic glycolysis identifies potential therapeutic targets in pancreatic cancer. Int. J. Biol. Sci. 17, 107–118. doi: 10.7150/ijbs.49243, PMID: 33390837 PMC7757027

